# Genomic Comparison of Non-Typhoidal *Salmonella enterica* Serovars Typhimurium, Enteritidis, Heidelberg, Hadar and Kentucky Isolates from Broiler Chickens

**DOI:** 10.1371/journal.pone.0128773

**Published:** 2015-06-17

**Authors:** Akhilesh S. Dhanani, Glenn Block, Ken Dewar, Vincenzo Forgetta, Edward Topp, Robert G. Beiko, Moussa S. Diarra

**Affiliations:** 1 Faculty of Computer Science, Dalhousie University, Halifax, Nova Scotia, B3H 4R2, Canada; 2 Pacific Agri-Food Research Center, Agriculture and Agri-Food Canada (AAFC), Agassiz, British Columbia, V0M 1A0, Canada; 3 McGill University and Genome Quebec Innovation Centre, Montreal, Quebec, H3A 1A4, Canada; 4 Lady Davis Institute for Medical Research, Montréal, Québec, H3T 1E2, Canada; 5 Southern Crop Protection and Food Research Centre, AAFC, London, Ontario, N5V 4T3, Canada; Institut National de la Recherche Agronomique, FRANCE

## Abstract

**Background:**

Non-typhoidal *Salmonella enterica* serovars, associated with different foods including poultry products, are important causes of bacterial gastroenteritis worldwide. The colonization of the chicken gut by *S*. *enterica* could result in the contamination of the environment and food chain. The aim of this study was to compare the genomes of 25 *S*. *enterica* serovars isolated from broiler chicken farms to assess their intra- and inter-genetic variability, with a focus on virulence and antibiotic resistance characteristics.

**Methodology/Principal Finding:**

The genomes of 25 *S*. *enterica* isolates covering five serovars (ten Typhimurium including three monophasic 4,[5],12:i:, four Enteritidis, three Hadar, four Heidelberg and four Kentucky) were sequenced. Most serovars were clustered in strongly supported phylogenetic clades, except for isolates of serovar Enteritidis that were scattered throughout the tree. Plasmids of varying sizes were detected in several isolates independently of serovars. Genes associated with the IncF plasmid and the IncI1 plasmid were identified in twelve and four isolates, respectively, while genes associated with the IncQ plasmid were found in one isolate. The presence of numerous genes associated with *Salmonella* pathogenicity islands (SPIs) was also confirmed. Components of the type III and IV secretion systems (T3SS and T4SS) varied in different isolates, which could explain in part, differences of their pathogenicity in humans and/or persistence in broilers. Conserved clusters of genes in the T3SS were detected that could be used in designing effective strategies (diagnostic, vaccination or treatments) to combat *Salmonella*. Antibiotic resistance genes (*CMY*, *aadA*, *ampC*, *florR*, *sul1*, *sulI*, *tetAB*, and *srtA*) and class I integrons were detected in resistant isolates while all isolates carried multidrug efflux pump systems regardless of their antibiotic susceptibility profile.

**Conclusions/Significance:**

This study showed that the predominant *Salmonella* serovars in broiler chickens harbor genes encoding adhesins, flagellar proteins, T3SS, iron acquisition systems, and antibiotic and metal resistance genes that may explain their pathogenicity, colonization ability and persistence in chicken. The existence of mobile genetic elements indicates that isolates from a given serovar could acquire and transfer genetic material. Conserved genes in the T3SS and T4SS that we have identified are promising candidates for identification of diagnostic, antimicrobial or vaccine targets for the control of *Salmonella *in broiler chickens.

## Introduction

Poultry is a major global source of protein, but infectious agents are significant concerns for both the industry and consumers. Many bacteria, including *Salmonella* species, can reside in healthy chickens without overt illness. The cecum is a primary site of *Salmonella* colonization in chickens and broiler chickens may carry this organism undetected into the abattoir at the time of slaughter, thereby representing a food-safety risk for consumers [1–3]. These bacteria have the potential to spread and contaminate farm workers and processing plants, food, and the natural environment.

Non-typhoidal *Salmonella* is one of the most common causes of bacterial gastroenteritis [4],[5]. *Salmonella* infection has been associated with many different foods, including poultry products [6], and some isolates have been found to be resistant to multiple antibiotics [7,8]. Despite the deployment of several control strategies such as integrated surveillance, multi-sectorial investigations, biosecurity and vaccination [9,10], *Salmonella* remains a significant challenge for the poultry industry. In Canada, salmonellosis was one of the three most common enteric bacterial diseases in 2010 with the serovars Enteritidis, Heidelberg and Typhimurium being the most frequently implicated in diseases [11]. Serovar Kentucky is a predominant serovar associated with poultry in Canada [11], but the factors driving its spread in commercial poultry production are not well understood. Better acid-stress responses of serovar Kentucky may confer a competitive advantage to this serovar over other *Salmonella* [12]. Furthermore, other differences may arise through the transfer of genetic elements such as plasmids harboring key virulence factors, which could play a role in the emergence of potential enteric pathogens such as *S*. *enterica* serovar Kentucky [13,14].

Several antimicrobial agents are used in poultry production for the treatment and prevention of diseases, which confer a selective advantage to resistant strains [15,16]. In some cases there is selection of hypermutable (high genome plasticity) strains having mutations in the DNA methyl mismatch repair (MMR) system [17,18]. A hypermutator *S*. *enterica* serovar Heidelberg strain with deletions in the *mutS* gene of the MMR system has been reported [18]. Such hypermutators have elevated rates of DNA recombination and acquisition of new virulence or antibiotic resistance genes [19].

The control of foodborne pathogens such as *Salmonella* is difficult because of their ability to survive during food production, processing, storage and improper cooking. Therefore, it is important to understand the ecology of *Salmonella* and the genetic variation of different serovars found in broiler chickens in order to design specific management practices to reduce risks associated with this pathogen. For example, understanding the genetic basis of intestinal colonization by different *Salmonella* serovars and their metabolic processes will provide insights into their survival mechanisms in the chicken gut, and may lead to the development of measures such as diagnostic, therapy or vaccination to prevent or limit their spread. Several molecular typing methods are used to differentiate *Salmonella* isolates, including multilocus variable-number tandem-repeat analysis, multilocus sequence typing or multiplex-PCR-based methods [20], and pulsed-field gel electrophoresis which is still considered the “gold standard” tool [21]. These methods are widely used in surveillance and epidemiological investigations but they also have value in studies on the ecology and population dynamics of *Salmonella* in diverse environments. However, these methods do not provide information on the whole genome, which is crucial for understanding the biology of *Salmonella*.

Whole-genome sequencing followed by comparative genome analysis is used to elucidate the evolutionary relationships of bacterial species, the genetic variation and plasticity underlying physiological differences, and to identify determinants of antibiotic resistance or virulence determinants [13,22,23]. The objective of the present study was to examine the complete genome sequences of representative isolates of *Salmonella enterica* serovars Typhimurium, Enteritidis, Hadar, Heidelberg and Kentucky recovered from commercial broiler chickens to provide a detailed comparative genomic analysis of these major serovars. These genome sequences were used to determine the phylogenetic relationships among members of the group, and to show how genomic variation between isolates may influence phenotypic traits such as antibiotic resistance, colonization, and virulence of previously characterised serovars [8] used in the present study.

## Materials and Methods

### Bacterial strains

The list of the 25 *Salmonella* isolates sequenced in this study is presented in Table 1. Isolates were collected in 2004 (eight isolates), 2005 (six isolates) and 2006 (11 isolates). All isolates were obtained from broiler chickens of different ages at 15 different commercial farms in British Columbia, Canada. In Canada, the top four *Salmonella* serovars implicated in humans salmonellosis are Typhimurium, Enteritidis, Hadar and Heidelberg, which justify the use of these serovars in this study. Serovar Kentucky also was included due to its widespread incidence in chickens, and its apparent non-virulence, to gain insights into its potential food safety risk. These 25 isolates, selected based on the prevalence of their respective serotypes, pulsotype and antibiotic susceptibility profiles, were previously described and partially characterised for their antimicrobial phenotype as well as partial information about their genotype, virulence and genetic diversity [8].

**Table 1:** List of the *Salmonella enterica* serovars from 15 different farms and their relevant characteristics as previously described [8].

Genome	Inventory #	*S*. *enterica* Serovar	Isolate	Sampling date	Farm	Age (days)	Resistance spectrum	Resistance genes	Virulence genes
1	3198	Typhimurium	ABBSB1113-1	26 June 2006	N	38	/	/	invA
2	1813	Typhimurium	SALF-297-3	6 Dec 2004	F	28	/	strA	invA-spvC
3	1893	Typhimurium	SALH-394-2	20 Dec 2004	H	12	AC,T,AM,F,C,Su,Te/Ax	blaCMY2-strA-aadA1	invA-spvC
5	1812	Typhimurium	SALF-297-2	6 Dec 2004	F	28	/	strA	invA-spvC
8	1803	Typhimurium	SALF-276-1	13 Nov 2004	F	12	AC,T,AM,F/Ax	strA	invA-spvC
9	3166	Typhimurium	ABB1162-2	27 Jun 2005	ND	ND	AC,T,AM,F,G,K,Su,Te	tetB-strA-sul1	invA
10	3148	Typhimurium	ABBSB1116-2	27 Jun 2006	O	36	/	/	invA
4	3176	4,[5],12:i:	ABBSB1205-1	2 Aug 2006	R	40	AC,T,AM,F/Ax	blaCMY2	invA-spvC
6	2008	4,[5],12:i:	SALI-436-2	3 Mar 2005	I	10	/	strA-aadA1	invA-spvC
7	3128	4,[5],12:i:	ABBSB1218-1	10 Aug 2006	S	42	AC,T,AM,F/Ax	blaCMY2-blaTEM	invA-spvC
11	3180	Enteritidis	ABBSB1004-1	9 May 2006	J	32	/	/	invA
12	3346	Enteritidis	ABB07-SB3071	28 Jun 2005	ND	ND	/	/	invA-spvC
13	3193	Enteritidis	ABBSB1050-2	29 May 2006	M	32/34	AC,T,AM,F	blaCMY2-blaTEM	invA
14	3172	Enteritidis	ABBSB1189-1	26 Jul 2006	M	33	AC,T,AM,F/Ax	blaCMY2-blaTEM	invA-spvC
15	3137	Hadar	ABB1048-1	27 Jun 2005	ND	ND	S,Te,	tetA-tetB-strA	invA
16	3187	Hadar	ABBSB1020-2	16 May 2006	L	42	AC,T,AX,AM,F,S,Su,Te	blaCMY2-blaTEM-tetA-tetB-aadA1-aadA2-sul1-strA	invA
17	3147	Hadar	ABBSB1121-1	28 Jun 2006	P	37	AM,T,Ax,Su,TSx,	sul1	invA
18	1770	Heidelberg	SALB-46	4 Oct 2004	B	10	AC,T,AP,F,Ax	strA	invA
19	1773	Heidelberg	SALB-159-4	25 Oct 2004	B	28	AC,T,AP,F,Ax	blaCMY2-strA	invA
20	3342	Heidelberg	ABB07-SB3031	28 Jun 2005	ND	ND	AC,T,AM,F,Ax	blaCMY2-blaTEM	invA
25	1768	Heidelberg	SALB-47-2	4 Oct 2004	B	10	AC,T,AM,F,Ax	strA	invA

* Resistant/intermediate (susceptible not listed), according to: AC = amoxicillin-clavulanic acid; T = ceftiofur; AX = ceftriaxone; AM = ampicillin; F = cefoxitin; G = gentamicin; K = kanamycin; C = chloramphenicol; Su = sulfisoxazole; Te = tetracycline S = streptomycin; TSx = trimethoprim-sulfamethoxazole. The 15 farms were designated B, C, F, H, J, I, K, L, M, N, O, P, R, S And ND (not determined).

### DNA extraction and genomic analysis

Genomic DNA from each of the 25 isolates was extracted using the GeneElute Bacterial Genomic DNA kit (Sigma-Aldrich, MO). The extracted DNA was stored in 1X TE buffer (pH 8.0) and quantified by using an Invitrogen Qubit 2.0 Fluorometer (Life Technologies). The quality of DNA was visualized after 1% agarose gel electrophoresis and then stored at -20°C until further use.

### Genome sequencing and assembly

Whole-genome shotgun library preparation and sequencing was performed using the Roche 454 GS system (GS-FLX Titanium), reagents and protocols for *S*. *enterica* serovar Enteritidis isolate ABBSB1189-1 resulting in a peak read length of ~375 nucleotides. The other 24 *Salmonella* isolates were sequenced using the Illumina HiSeq platform following manufacturer’s protocols resulting in 200-nucleotide paired-end reads. Illumina sequence reads were quality filtered using Sickle [24] using a minimum length criterion of 50 nt and a minimum quality score of 30. Sequences that satisfied these criteria were then assembled using Velvet version 1.2.08 with a range of *k*-values between 43 and 51. The resulting n50 values ranged from 25,511 to 485,223 (S1A Fig): although it was not optimal in every case, *k* = 51 produced the best average n50 value (249,713). Contigs from these genomes were reordered using Mauve [25] and aligned using the finished genome of *S*. *enterica* serovar Typhimurium strain UK-1 [26]. This finished genome was chosen based on a high degree of gene order conservation with respect to our serovars: for example, this reference genome aligned to our genome #1 (S. Typhimurium isolate ABBSB1113-1) with only two translocated blocks of genes identified. Contigs that failed to match this reference (and were consequently not reordered by Mauve) were added to the end of the genome file.

Plasmid identification in the sequenced isolates was carried out by combining two distinct lines of evidence: average depth of coverage and matching of assembled contigs to 114 known plasmids from our Roche/454 reference and other published *Escherichia* and *Salmonella* genomes. Sequenced contigs were tiled against reference plasmids based on the identification of consecutive genes that were homologous between contigs and plasmids. Sets of contigs that covered a majority of reference plasmid genes were proposed to be plasmids in the newly sequenced genome. This procedure of assembling contigs into inferred plasmids was verified using read depth: nearly all plasmid-associated contigs had read depths that were similar to one another, and above the range of chromosomal contigs (with the exception of multi-copy ribosomal genes). Read depth was also used to infer the presence of plasmids whose genes did not tile well against reference plasmids: any non-ribosomal contigs or set of contigs with read depth above the range of chromosomal contigs and not already part of an inferred plasmid was considered a candidate plasmid. These candidate plasmids were typically small (a few thousand nucleotides in length), present in read depths at least 10 times those of the chromosomal genes, and consisted only of genes necessary for plasmid replication.

### Annotation and comparative analysis

Annotation of assembled genomes was performed with the RAST server [27] in May 2013. Errors and frameshifts were corrected automatically by the server. RAST annotations were retrieved in GenBank format. For comparative purposes, we included in the analysis a total of 55 strains of *E*. *coli*, the recently published genome of *E*. *fergusonii* [13], and 30 strains of *Salmonella* including 29 *S*. *enterica* and one *S*. *bongori* (see S2 Table for a complete list of included strains). These reference genomes were obtained from NCBI RefSeq on December 6, 2013. The predicted protein sequences of all these reference strains were combined with those of our 25 new isolates into a single file, and clusters were subsequently constructed using UCLUST version 3.0.617 [28]. Protein sequences were sorted in decreasing order of length. Clusters were built by identifying seed sequences that were dissimilar from all sequences seen before, then adding to those clusters any protein that was at least 90% identical in its amino-acid sequence. The complete set of annotations for proteins in a given cluster was merged. Each cluster also served as the basis for a phylogenetic profile [29,30], which records the presence or absence of gene clusters for proteins in each genome. Clusters predicted to be present on both plasmids and chromosomes were inspected in greater depth, and corrected where appropriate. Due to the stringency used in the alignment, some clusters were found to be associated with plasmids or chromosomes in different isolates. These sequences were then manually curated against the reference plasmid database and corrected accordingly.

### Phylogenetic analysis

Phylogenetic analysis of the sequenced *Salmonella* isolates was carried out using a reference set comprising all complete and draft genomes of the genera *Escherichia* and *Salmonella* that were available from NCBI RefSeq on December 6, 2013. The completed genome set included 63 *E*. *coli*, one *E*. *fergusonii*, and 45 *Salmonella*. The draft genome set included another 700 *Escherichia* and 283 *Salmonella* genome projects, of which 682 and 278, respectively, had associated sequences. Protein sequences predicted to be components of the large and small ribosomal subunits were extracted from two genomes, *Escherichia* sp. 1_1_43 and *Salmonella enterica* subsp. Enterica serovar Typhi str. Ty2, and used as queries against the predicted proteins from genome sequences in their respective genera. The ribosomal proteins were used because they are most likely to be vertically inherited and minimally confounded by paralogy [31]; including more genes would have yielded more SNP information but raised the risk of confounding signals due to lateral gene transfer. Homology searching was performed using BLASTP 2.2.23+, with a maximum e-value threshold of 10^-30^. Matching protein sequences were then mapped back to their corresponding genes in both reference and our new genomes, and the two sets were merged into nucleotide sequence files. To build the concatenated alignment, we required that each genome contain at least 80% of all loci, and that each locus be present in at least 80% of all taxa. This yielded a final set of 980 genomes and 41 ribosomal genes. Concatenated alignments were trimmed with Gblocks [32] with default settings. Identical sequences in the set were then reduced to a single representative sequence, which yielded a total of 637 unique DNA sequences.

An initial tree of these sequences was constructed using FastTree version 2.1.0 SSE3 [33] with a GTR model of amino acid substitution and the settings “-slow-spr 10-sprlength 20-slownni” to conduct a more detailed search. The resulting tree was then used as a starting tree for RAxML version 7.2.5-mpi [34], with default settings and a GTR model with a discrete gamma model of rate variation. One hundred bootstrap replicates were performed to estimate the support for each inferred relationship in the tree. From this tree, a smaller set of 40 taxa was selected, comprising all our 25 newly sequenced *Salmonella* genomes, representative sequences of *E*. *coli*, *E*. *albertii* and *E*. *fergusonii*, and 12 *Salmonella* strains closely related to the sequenced isolates (as inferred from the larger tree).

### Identification of functional gene classes

The 90% identity protein clusters constructed above were searched to identify sets of candidate virulence proteins. In each case, the phylogenetic profiles of all associated proteins were compared to identify groups of clusters with similar profiles. For all functional classes considered, we also determined whether proteins in each cluster were predicted to reside on plasmids or on the bacterial chromosome. For functional groups with many members, heatmaps showing the phylogenetic profiles of all implicated clusters were constructed using the R statistical language [35].

#### Adherence factors

Adherence-associated clusters were identified among the newly sequenced isolates using VFDB (Virulence Factor Database) which included fimbrial and non-fimbrial associated adherence factors [36].

#### Toxins and flagella

The clusters associated with these functional groups among newly sequenced isolates were screened using specific keywords (i.e. gene names) and reference literature [37–39].

#### Type III secretion system (T3SS)

To identify the T3SS-associated proteins, we downloaded all protein sequences in T3DB version v201110 [40] and compared them to cluster-associated proteins using BLASTP 2.2.23+ with a maximum e-value threshold of 10^-5^. Clusters matching sequences from T3DB were included in the T3SS data set.

#### Type IV secretion system (T4SS)

The T4SS associated proteins were acquired from the SecReT4 database [41], and compared to cluster-associated proteins as above.

#### Iron resistance proteins

Acquisition of iron plays an important role in the survival and pathogenesis of most bacteria. The relatedness of *Salmonella* serovars according to the presence of iron transport- and utilization-related genes were determined [42].

#### General virulence-associated proteins

We obtained a broader view of virulence proteins by comparing cluster-associated proteins with those from MVirDB [43] using BLAST as above.

#### Hypermutatibility

The methyl-directed DNA mismatch repair (MMR) system protects the bacterial genome from acquiring new phenotypes by both mutation and recombination. Deficiency of the *mut*HLS regulon or other methyl-directed mismatch repair proteins increases the mutation frequency and the rate at which two dissimilar sequences recombine [44]. The protein clusters associated with these functions were investigated in our newly sequenced serovars to identify strains prone to rapid genetic and phenotypic changes.

We used read mapping to examine cases where functional genes were proposed to be absent in some isolates based on assembly and annotation. Homology searches were performed using an appropriate reference gene from RefSeq gene records, and the raw, unassembled reads from each reference genome. Comparisons were performed using BLASTN version 2.2.23+ [45] with default parameters, except for a threshold e-value of 10^-5^ and a maximum of 50,000 results returned.

### Nucleotide Sequence Accession Numbers

This Whole Genome Shotgun project has been deposited at DDBJ/EMBL/GenBank under the BioProject ID number is: PRJNA273513. The accession numbers are: LAPA00000000, LAPB00000000, LAPC00000000, LAPD00000000, LAPE00000000, LAPF00000000, LAPG00000000, LAPH00000000, LAPI00000000, LAPJ00000000; LAPK00000000, LAPL00000000, LAPM00000000, LAPN00000000, LAPO00000000, LAPP00000000, LAPQ00000000, LAOS00000000, LAOT00000000, LAOU00000000, LAOV00000000, LAOW00000000, LAOX00000000, LAOY00000000 and LAOZ00000000.

## Results

### Sequencing, assembly and annotation

Illumina sequencing of the 24 isolates yielded in excess of 331 million reads and 66 billion nucleotides of DNA sequence. The 454 sequencing of the isolate ABBSB1189-1 of serovar Enteritidis produced 569,003 reads and 179 million nucleotides in total. There was a weak relationship between average sequence quality and Velvet n50 score (S1B Fig), with four genomes notably having n50 < 60,000. No combination of quality-filtering criteria and *k* variation substantially increased the n50 of these genomes. Three of the four genomes belonged to serovar Kentucky, while the last was an isolate of serovar Heidelberg. Genomes sequenced on the Illumina platform had between 111 and 451 contigs, and ranged in predicted size from 4.68 to 5.08 megabases (S1 Table). The 454-sequenced genome had 71 contigs and an estimated size of 4.96 megabases. All genomes were very similar in their % G+C (52.17% to 52.46%) and gene density (one gene every 990 to 1021 bases in the genome, on average). The number of annotated protein-coding genes varied between 4599 and 5067, in line with the variation in genome size. Genome statistics from the four genomes with very low n50 scores were consistent with those from the other 21 (S1 Table), suggesting that poor assembly did not substantially impact the calling of genes.

### Gene/Protein clusters

The 548,535 predicted proteins from 111 genomes produced a total of 18,619 clusters at 90% amino acid identity or greater (S2 Table, S1 File). Of these, 9175 were restricted to *E*. *coli* and discarded. Of the remaining 9444 clusters, 9423 (99.8%) were found in the 25 sequenced isolates of *Salmonella* (S2 Fig), with 38.2%, and 28.6% of these clusters present in all 25 genomes (i.e., the core genome), and in only one isolate, respectively. The conserved core consisted of 3603 proteins, equivalent to approximately 75% of any given *Salmonella* genome in the set.

### Phylogenetic analysis

**Fig 1:**
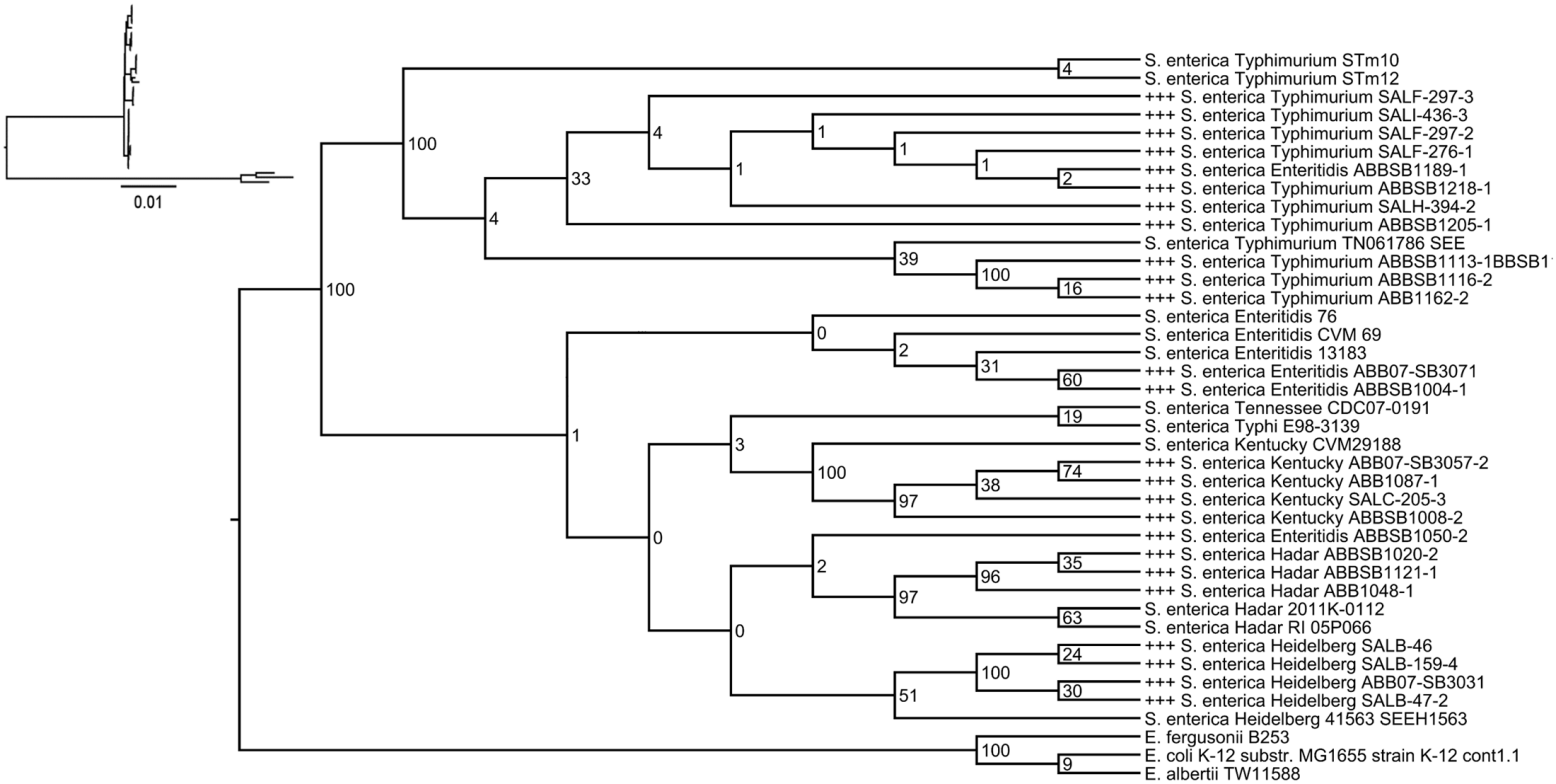
Projected phylogeny of isolate genomes extracted from the full tree of *Escherichia* and *Salmonella* isolates (see S2 Fig). Numbers at internal nodes correspond to bootstrap values supporting the indicated clades in the full tree. “+++” indicates newly sequenced isolates. Inset: pruned tree shown with branch lengths corresponding to substitutions per site.

The genome phylogeny built from 637 genomes based on concatenated ribosomal genes separated *Salmonella* from *Escherichia*, and we fixed the root of the tree between these genera (S3 Fig, S2 File). Apart from *S*. *bongori* and *S*. *enterica* serovar Salamae, the tree of *Salmonella* isolates is characterized by very short branches, typically with < 1 substitution per 1000 sites. In spite of this, major serovars including Typhimurium, Typhi, Heidelberg, Hadar, Montevideo, Kentucky and Enteritidis (with the exception of isolate ABBSB1189-1, which branches within Typhimurium) are clades in the tree, typically with bootstrap support near or equal to 100. A notable exception to this pattern is the serovar Newport, which is known to be polyphyletic [46] and forms two distinct clades in our tree. The reduced phylogeny (Fig 1) highlights the very strong support for serotype (serovar) clades except of Enteritidis, and minimal support for relationships that group different isolates or subsets of isolates (4,[5],12:i:) within serotypes. Partial exceptions to this pattern include isolates from serovars Kentucky and Hadar, which have higher support for within-serovar relationships. With the exception of an isolate of serovar Enteritidis (ABBSB1189-1), all newly sequenced isolates were in clades with earlier isolates of the same serovar.

### Functional profiling of sequenced isolates

We examined the predicted gene functions of all 25 genomes in depth to characterize the distribution of virulence factors and other attributes related to pathogenicity and survival.

#### Adherence factors

The largest numbers of gene clusters associated with adhesion were observed in the studied broiler *Salmonella* compared to the chosen reference *Salmonella*. All isolates apart from three Typhimurium carried more than 92 gene clusters with the largest number (99 clusters) observed in serovar Heidelberg.

**Fig 2:**
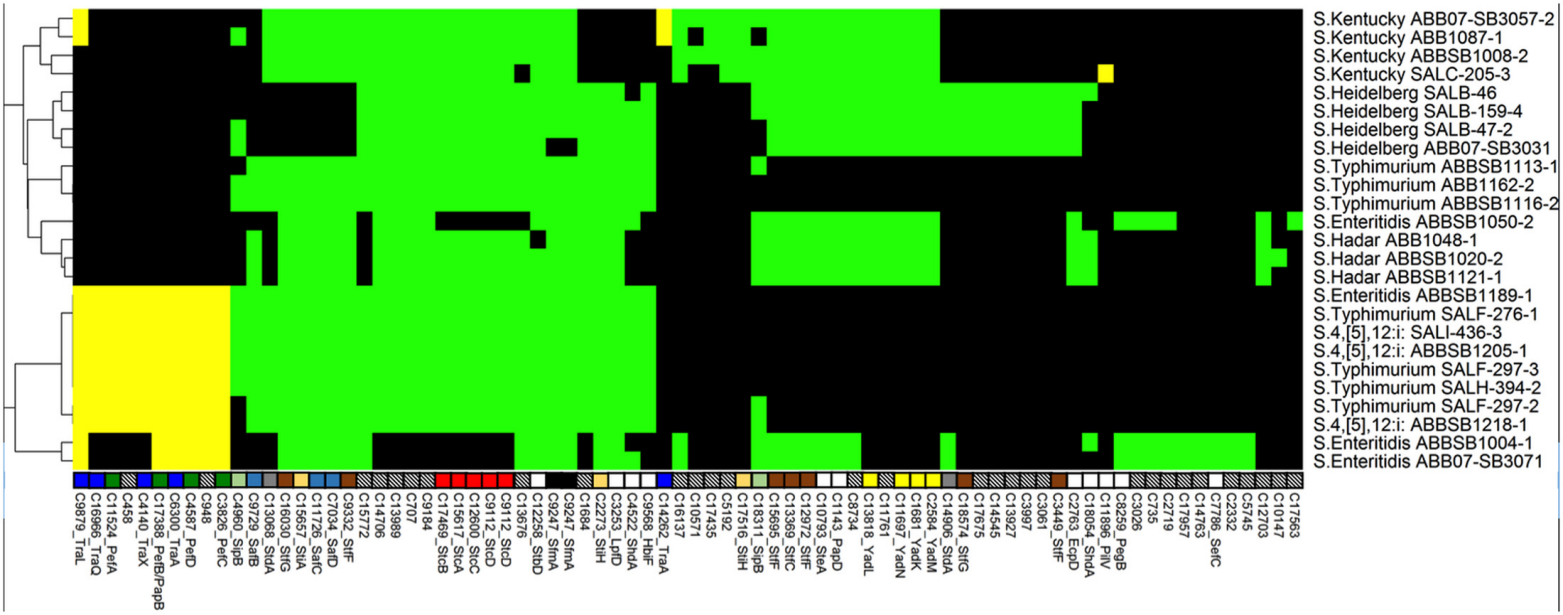
Heatmap showing the distribution of 78 adhesion-associated proteins across the 25 newly sequenced *Salmonella* genomes. Another 54 ubiquitous proteins are not shown. Column labels indicate unique cluster IDs (see S1 File), and gene names where appropriate. Black cells = absence of a gene from a given strain, green cells = probable chromosomal, yellow = probable plasmid. The tree on the left-hand side of the figure shows a hierarchical complete-linkage clustering of the profiles based on Euclidean distance. Multiple genes affiliated with the same operon are identified by coloured square boxes above the cluster legends. Empty boxes represent a single, independent cluster and boxes with patterns represent clusters without annotated gene names.

The fimbrial operons *fim*AICDHF (type 1 fimbriae), *lpf*ABCDE (long polar fimbriae), *agf*/*csg*ABCEFG (thin aggregative fimbriae), *stf*ACDEFG and *sth*ABCDE were present in all newly sequenced *Salmonella* genomes (Fig 2). Only eight isolates including seven isolates of the serovar Typhimurium and its three monophasic 4,[5],12:i: as well as one isolate of the serovar Enteritidis had all four genes needed to form structural plasmid-encoded fimbriae (*pef*ABCD). An incomplete operon *stb*BCD (missing the *stb*A gene) was found in all *Salmonella* assemblies, and *stbA* was detected in all the isolates on read mapping. Furthermore all our three isolates of the serovar Hadar and *S*. Enteritidis ABBSB1050-2 have *std*B alone rather than the *std*AB operon; however, a truncated *stdA* gene was found adjacent to *stdB* based on read mapping. In addition, operon *stc*ABCD was also present in all isolates with the exception of three isolates of serovar Enteritidis. Among the four serovars Enteritidis, only the isolate ABBSB1189-1 (which branches with Typhimurium in the reference phylogeny) contained the *pef* and *stc* fimbriae-associated operons. Discrete clusters containing the *sti*, *saf*, *stj* and *bcf* operons were found in *Salmonella* genomes but none of the isolates had the complete set of genes required to form structural fimbriae. Among the non-fimbrial adhesion factors, genes encoding MisL, RatB, ShdA and SinH proteins were present in all studied *Salmonella* genomes, except ShdA which was absent from isolates of the serovar Kentucky. Comparison of a reference ShdA protein against the raw reads of serovar Kentucky isolates revealed missing sections of the corresponding gene of this protein.

#### Toxins

Hemolysin E was completely absent from all 25 *Salmonella* genomes but plasmid-borne hemolysin F genes (*hly*F) were present in two isolates of serovar Kentucky. However, all 25 sequenced isolates were found to carry the putative hemolysin and hemolysin-coagulated proteins. Additionally, all isolates harboured the hemolysin expression regulator Hha. Among other toxins, colicins (narrow-spectrum pore-forming bacteriocins) were observed in all 25 *Salmonella* genomes. Both colicin Ia and the corresponding immunity protein were present in an isolate of serovar Kentucky (ABB1087-1) and three isolates of serovar Heidelberg (SALB-46, SALB-159-4 and SALB-47-2). All 25 isolates also harboured gene clusters encoding the colicin-binding catecholate siderophore receptor and its uptake protein as well as the colicin V production protein. Additionally, all four isolates of serovar Kentucky contained the gene encoding the colicin E7 immunity protein, but the corresponding colicin gene was not detected. The operon *yej*ABEF, which encodes a putative ATP-binding cassette (ABC) transporter contributing to the virulence of *Salmonella*, was detected in all 25 newly sequenced *Salmonella* genomes.

#### Type 3 secretion system

**Fig 3:**
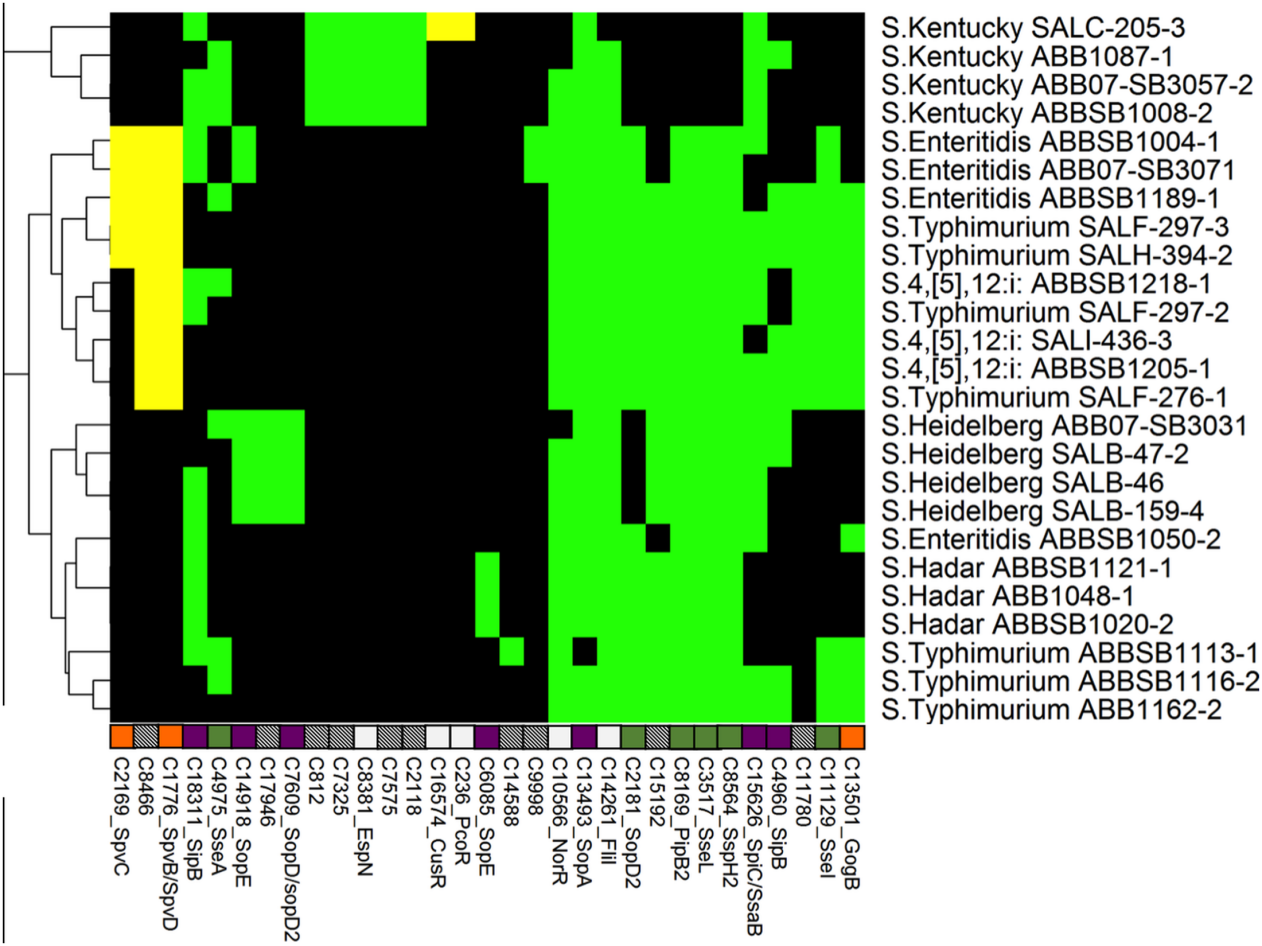
Heatmap showing the distribution of 31 type 3 secretion system proteins. Another 111 ubiquitous proteins are not shown. Labels, colors and clustering are consistent with Fig 2.

Among the T3SS-associated genes, *invB*, a chaperone for SopE, a bacteriophage-encoded effector, was detected on the SPI-1 of all 25 *Salmonella* genomes according to read mapping. InvB is required for translocation of SopE through T3SS-1 [47]. However, other SPI-1-associated effectors SipA, translocase (SipB, SipC and SipD), SptP as well as non-SPI-1-associated effectors AvrA, SopA, SopB, SopD, and SopE2 were present in all the genomes. In general, all other chaperones, regulators and secretion apparatus-linked proteins of T3SS1 were detected in all 25 newly sequenced *Salmonella* genomes (Fig 3).

Out of the 25 newly sequenced genomes, the four isolates of serovar Kentucky lacked full-length SPI-2 associated genes *sopD2*, *pipB2*, *sspH2* and *sseI* (*srfH)*, which were found in all other serovars except *sseI*, which was also absent from all isolates of serovar Heidelberg, Hadar and *S*. Enteritidis ABBSB1050-2. The SPI2-encoded effector SpiC (SsaB) has a significant role in the intracellular formation of the *Salmonella*-containing vacuole (SCV) within lysosomes. This effector was present in all 18 isolates (eight Typhimurium, all four Kentucky, all four Heidelberg and two Enteritidis) but absent from seven isolates (all three Hadar, two Typhimurium and two Enteritidis) (Fig 3). The presence of these SPI2-associated effectors indicates the virulence potential of isolates harboring them; however, deletions observed in *sopD2* of Kentucky might compromise their virulence. Ramos-Morales et al. summarized 26 other effectors associated with T3SSs [48] known to have no significant impact on the virulence of *Salmonella in vivo*. Of these effectors, SrfJ was present in Hadar, Typhimurium, and Enteritidis; fragments of SspH1 were found in all isolates; while SseK (1–3), and Ste (A-C) were completely absent from all our 25 newly sequenced *Salmonella* genomes.

#### Type 4 secretion system

**Fig 4:**
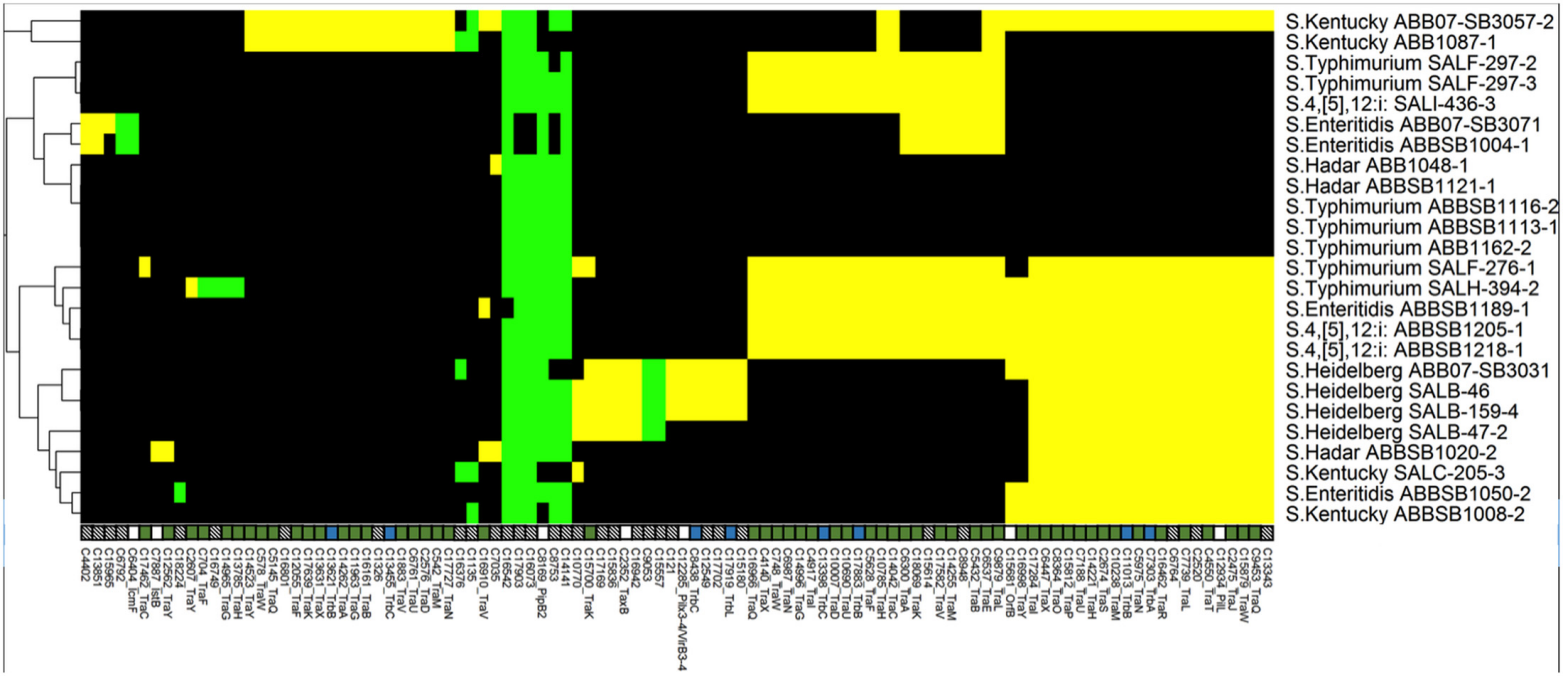
Heatmap showing the distribution of 102 T4SS proteins. Another 17 ubiquitous proteins are not shown. Labels, colors and clustering are consistent with Fig 2.

Several T4SS-associated gene clusters were identified in the 25 studied *Salmonella* genomes (Fig 4). The identified T4SS genes, which were associated with putative plasmids (Fig 4) among the isolates, showed a heterogeneous distribution. The type IV pilin biogenesis gene *hofC* and the Type IV fimbrial assembly gene encoding the PilC protein were found in all 25 isolates. The type IV secretion conjugal transfer ATPase *pilx3-4* (*virB3-4*) gene was found only in three of the four isolates of serovar Heidelberg. Type IVB thin pilus genes *pilM*, *pilP* and *pilO*, which encode putative IncI1 conjugal transfer pilus assembly proteins, were identified in all 25 studied *Salmonella* isolates. Genes encoding the F-like transfer systems associated with plasmid propagation and maintenance were identified in 14 isolates. All 14 isolates have the essential structural and functional F-like transfer system genes, however the six isolates *S*. Enteritidis ABBSB1189-1, *S*. Kentucky ABB07-SB3057-2, *S*. Typhimurium (SALH-394-2 and SALF-276-1) and *S*. 4,[5],12:i (ABBSB1205-1 and ABBSB1218-1) also carried a variety of *Tra* operon-associated genes.

#### Flagella

**Fig 5:**
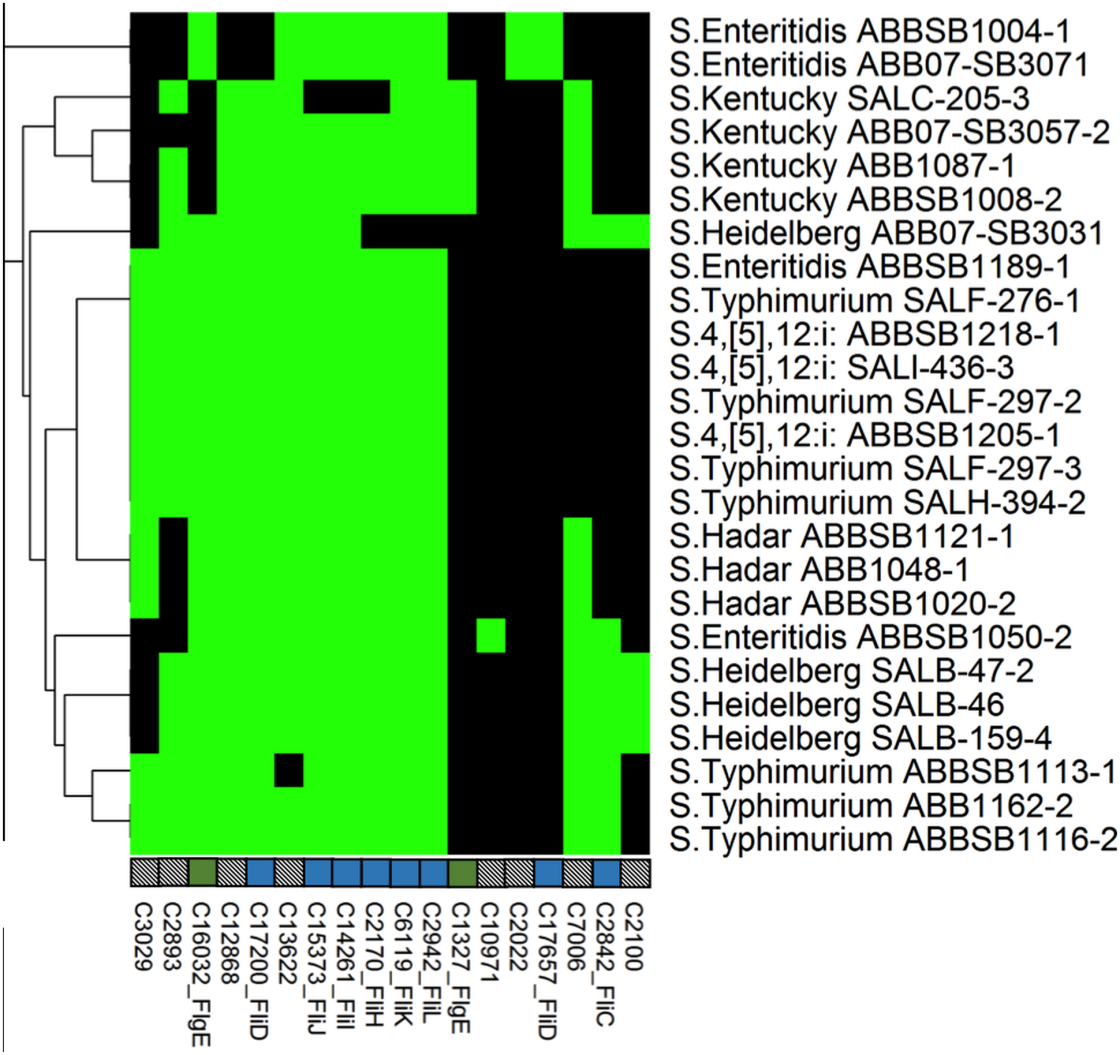
Heatmap showing the distribution of 18 flagellar proteins. Another 40 ubiquitous proteins are not shown. Labels, colors and clustering are consistent with Fig 2 (no flagellar proteins were predicted to be plasmid associated).

Flagella confer mobility to bacterial. In general, all 25 isolates had a cluster profile similar to the selected reference *Salmonella*. In total, 58 flagella-associated proteins were found in the newly sequenced *Salmonella* genomes; forty of these, comprising most structural components of the flagellum, were found in all serovars (Fig 5). FlgE, a flagellar hook protein, was clustered in two separate groups, one found only in isolates of serovar Kentucky with a 22 amino-acid deletion, while the second group was present in all other isolates. However, a Heidelberg isolate harboured a gene encoding a truncated version of the protein with deletions at the N-terminus and the conserved C-terminus. Additionally, the same two isolates of serovar Enteritidis were carrying an identical in length but dissimilar in sequence (split into two clusters) copy of *fli*D and Phase-1 flagellin compared to all other 23 studied isolates (Fig 5). The structural flagellin gene *fli*C was present in all isolates of serovars Typhimurium and Kentucky (full length) and in all isolates of serovars Heidelberg, Hadar and Enteritidis (partial length).

#### Iron uptake and assimilation

**Fig 6:**
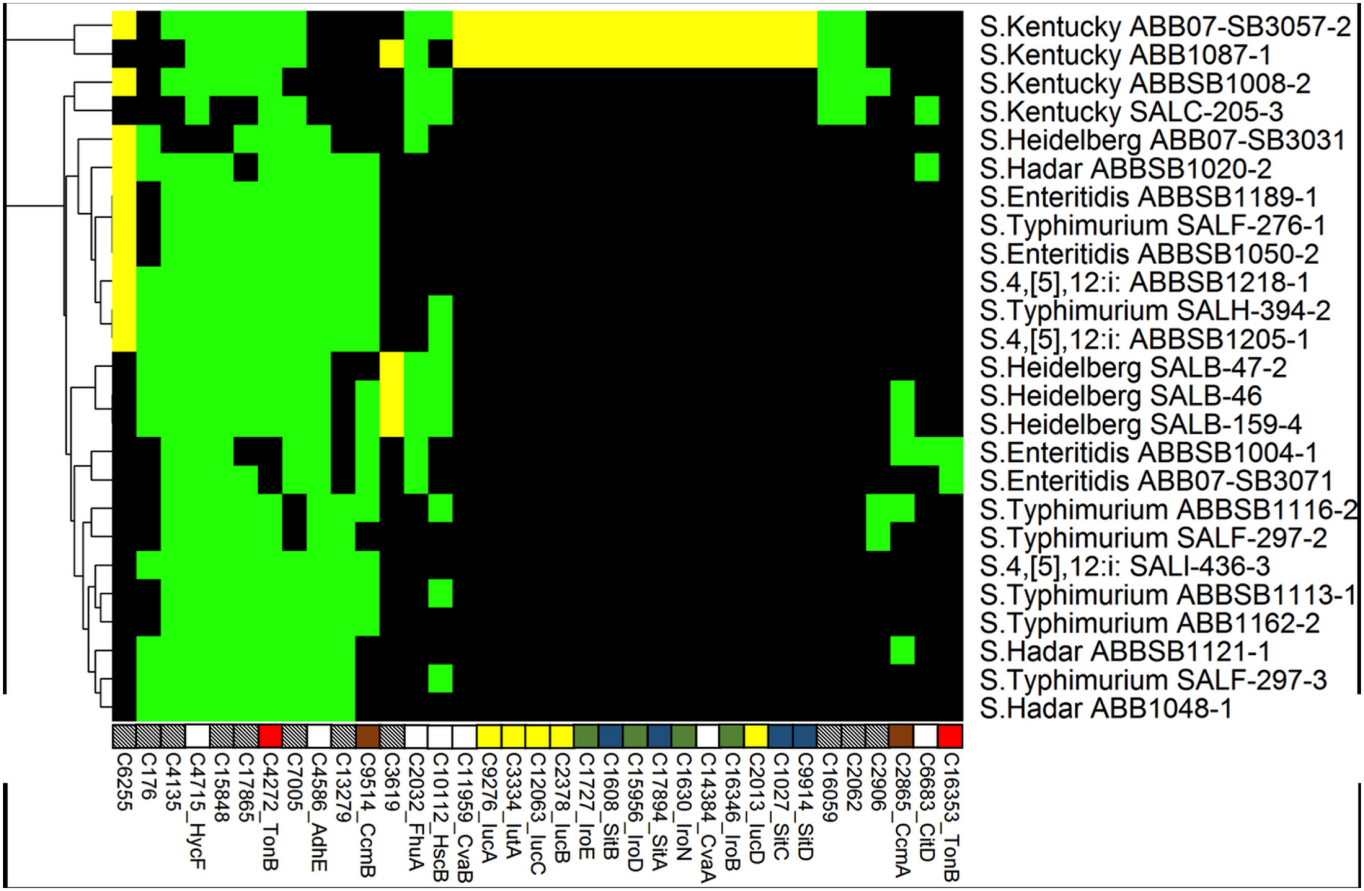
Heatmap showing the distribution of 35 iron resistance proteins. Another 87 ubiquitous proteins are not shown. Labels, colors and clustering are consistent with Fig 2.

Genes encoding the FeoA and FeoB proteins (involved in uptake of soluble Fe^2+^) were present in all 25 *Salmonella*. Siderophores are Fe^3+^-chelators secreted by most pathogenic bacteria including *Salmonella* in a response to iron-limiting environments. In this study, all isolates of *Salmonella* were equipped with the catecholate siderophore enterobactin synthesis and utilization genes including the iron-siderophore outer membrane receptors *cirA*, *fepA*, and transporters *fepBCDEG*. In addition, two of the four isolates of serovar Kentucky (ABB1087-1 and ABB07-SB3057-2) harbored the siderophore salmochelin (a C-glucosylated enterobactin produced by *Salmonella* species, uropathogenic and avian pathogenic *E*. *coli* strains, and certain *Klebsiella* strains) associated proteins *iroN* and *iroBCDE* (Fig 6) These same two isolates of serovar Kentucky carried the iron acquisition system comprising the siderophore aerobactin biosynthesis (*iucABCD*) and a high-affinity outer membrane ferric aerobactin receptor (*iutA*) which was seen in none of the reference genomes of *Salmonella*. Furthermore, these two serovar Kentucky isolates also carried the putative iron transport system operon *sitABCD*. The ferric-siderophore transport proteins TonB, ExbB and ExbD, as well as the hydroxamate siderophore transport genes *fhuBCD* were detected in all studied isolates. The ferrichrome outer membrane transporter-ferrichrome-iron receptor (FhuA) was grouped in two separate clusters based on differences in N-terminal sequence. Among the functional enzymes, none of the isolates of serovar Kentucky harbored the iron-dependent alcohol dehydrogenase.

#### Antimicrobial resistance and cell defence

**Fig 7:**
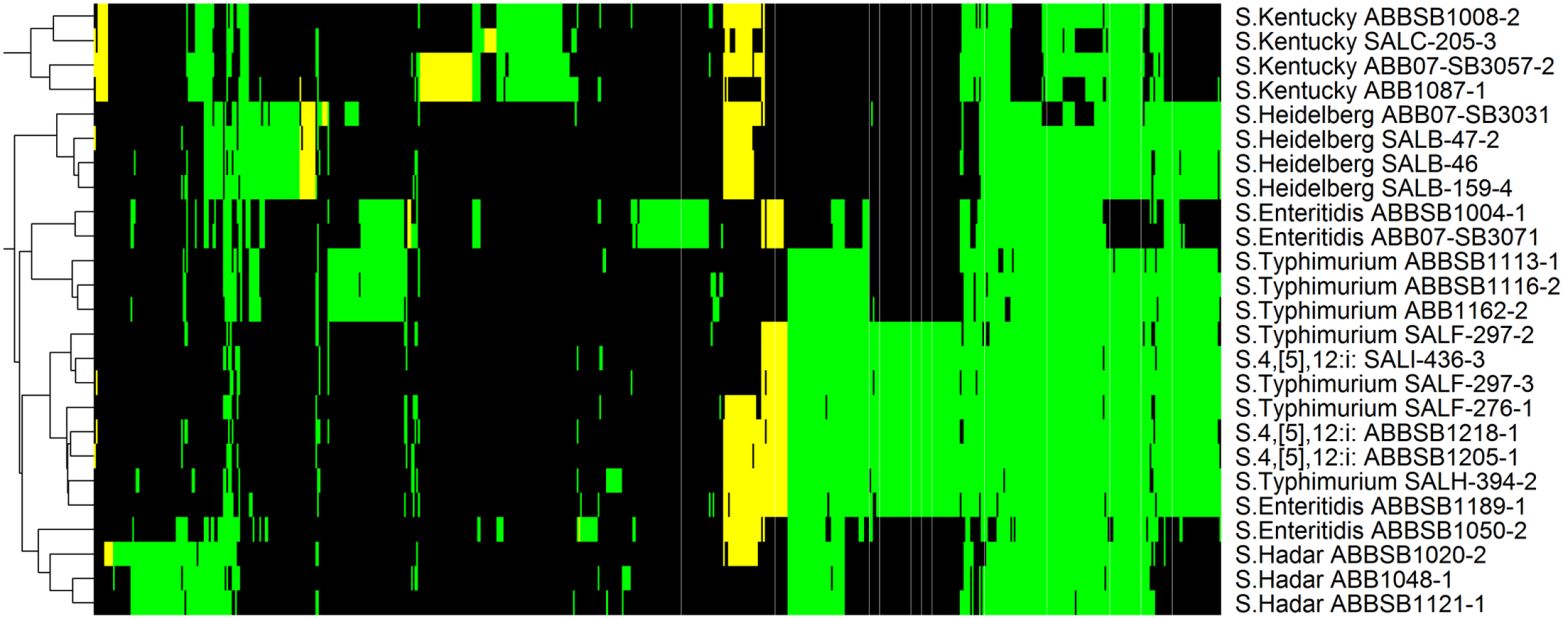
Heatmap of 647 proteins matching to the MVirDB database. Another 784 ubiquitous proteins are not shown. Colors are consistent with Fig 2; column labels are shown in S4 Fig.

We compared antibiotic and metal resistance genes of the studied *Salmonella* genomes to identify different mechanisms of resistance (Fig 7, S4 Fig). A total of 181 protein clusters were found to be associated with defence and resistance attributes based on matches to MVirDB. Among them, 152 clusters were found in our newly sequenced *Salmonella* genomes, with 101 of these present in all 25 *Salmonella* isolates. Several identified efflux systems are known to pump a wide range of toxic molecules including antibiotics, metals, detergents and bile salts, out of the bacterial cell. Among them the AcrABC and MdtC systems of the resistance-modulated cell division (RND) family efflux, as well as the EmrABC operon encoding the major facilitator superfamily (MFS) multidrug efflux pump were found in all 25 isolates. Two other multiple antibiotic-resistance (mar) regulons, *marR* (DNA-binding transcriptional repressor) and the *marABC* system, known to confer resistance to various antibiotics such as cephalosporins and tetracycline, were also found in all studied isolates. Apart from the isolate SALH-394-2 of serovar Typhimurium which harbored the *floR* gene, no chloramphenicol resistance gene cluster was found in the studied *Salmonella* genomes. The sulfonamide resistance gene dihydropteroate synthase type-2 (*sul2)* was also detected in this isolate. The Zn-dependent hydrolase β-lactamase, and the streptomycin 3''-O-adenyltransferase genes were identified in all 25 isolates while the AmpC-like beta-lactamase (*bla*_CMY-2_) was found in 13 of them (four Typhimurium, two Enteritidis, all four Heidelberg, one Hadar and two Kentucky). The transposon *Tn21* resolvase TnpR and the tetracycline efflux *tetA* gene were found in all three isolates of serovar Hadar, however only the dihydropteroate synthase *sul1* gene known to be associated with the Class 1 integron, the aminoglycoside 3-*N*-acetyltransferase (*aac3-VI*) and the aminoglycoside adenyltransferase gene (*aadA*) were detected in one isolate (ABBSB1020-2) of this serovar.

Interestingly, *S*. Kentucky SALC-205-3 (see hypermutation section below) carried four unique gene clusters linked to copper and silver resistance: the copper/silver efflux system membrane component proteins CusA/SilA, CusB//SilB and CusC//SilC, and the PcoABC operon for copper resistance. These clusters were absent from all the reference *Salmonella* genomes used but were present in a few *E*. *coli* genomes. The isolates of serovar Heidelberg have three distinctive clusters, two related to arsenic resistance and one associated with fosfomycin resistance. Fosfomycin is a broad-spectrum antibiotic used to treat lower urinary tract infections especially those caused by extended-spectrum β-lactamase-producing organisms. Surprisingly, this cluster was completely absent from reference *E*. *coli* genomes and present only on the chromosome of the *S*. Heidelberg genomes. Other discrete clusters found in *S*. Hadar include metabolite transporters, resolvases and tetracycline resistance proteins. Mercury-resistance operons were completely absent from all 25 *Salmonella* genomes, however genes linked to biofilm formation, bile salt resistance and acid tolerance were found in all of them. The largest number of these clusters was associated with *S*. Kentucky which includes some unique clusters associated with arsenic resistance.

### Plasmids

All sequenced genomes had at least one inferred plasmid based on the criteria described in the Materials and Methods section above (Table 2, S3 Table). Twelve of the sequenced genomes included genes associated with plasmid IncF, while 14 had genes associated with plasmid IncI1 and one harbored plasmid IncQ. Genes coding for plasmid conjugative mobilization and transfer factors, kappa-fimbriae, actin-ADP-ribosyltransferase, toxins SpvB, SpvD, and VapBC, invasins, virulence factor transcriptional activators and the streptomycin 3''-O-adenyltransferase which confers resistance to some aminoglycoside antibiotics were often associated with predicted IncF-plasmids. In *S*. Kentucky ABB1087, the IncF plasmid was also predicted to carry the RND efflux system conferring multidrug resistance, which comprises the macrolide-specific efflux gene *macA*, the aminoglycoside 3'-phosphotransferase and the tetracycline efflux gene *tetA*. Read mapping suggested the presence of full-length *macA* in all 25 strains, but *tetA* in only two Kentucky strains. In addition, *S*. Kentucky ABB07-SB3057-2 carries the outer membrane siderophore receptor *iroN* and its associated genes *iroBCDE*, as well as *sitABCD* genes associated with manganese transport and *iucAB* encoding genes for siderophore aerobactin biosynthesis and utilization. The quaternary ammonium compound resistance protein SugE and β-lactamase genes were found on the IncI plasmid. Five isolates showed no evidence of Inc plasmids.

**Table 2:** Overview of the predicted plasmids in 25 *Salmonella enterica* isolates.

Serovar (genome #)	Plasmid name	Incompatibility group	Size (nt)	# ORFs	# Contigs	Average Read Depth
Typhimurium ABBSB1113-1 (#1)	pSB1113-1_1		4180	5	1	2652
	pSB1113-1_2		3157	3	1	3512
Typhimurium SALF-297-3 (#2)	pSALF-297-3_1	IncF	90,907	130	4	85
	pSALF-297-3_2		2095	1	1	1763
	pSALF-297-3_3		4074	3	1	1245
Typhimurium SALH-394-2 (#3)	pSALH-394-2_1	IncI1	99,953	109	6	259
	pSALH-394-2_2	IncF	91,741	133	5	185
	pSALH-394-2_3		5444	9	1	4337
Typhimurium 4,[5],12:i: ABBSB1205-1 (#4)	pSB1205-1_1	IncF	98,899	187	7	402
	pSB1205-1_2	IncI1	86,781	95	2	697
Typhimurium SALF-297-2 (#5)	pSALF-297-2_1	IncF	186,410	246	5	311
	pSALF-297-2_2		2095	1	1	8518
	pSALF-297-2_3		4074	3	1	5910
Typhimurium 4,[5],12:i: SALI-436-3 (#6)	pSALI-436-3_1	IncF	93,491	134	4	158
	pSALI-436-3_2		4074	3	1	2711
	pSALI-436-3_3		2095	1	1	4775
Typhimurium 4,[5],12:i: ABBSB1218-1 (#7)	pSB1218-1_1	IncF	81,267	113	4	559
	pSB1218-1_2	IncI1	86,781	94	2	1075
Typhimurium SALF-276-1 (#8)	pSALF-276-1_1	IncF	89,201	131	4	286
	pSALF-276-1_2	IncI1	88,870	93	4	611
Typhimurium ABB1162-2 (#9)	pABB1162-2_1		3163	4	1	8606
	pABB1162-2_2		4184	5	1	6168
	pABB1162-2_3		21,176	27	2	367
Typhimurium ABBSB1116-2 (#10)	pSB1116-2		3137	3	1	2882
Enteritidis ABBSB1004-1 (#11)	pSB1004-1_1	IncF	57,522	88	1	353
	pSB1004-1_2		6379	5	1	580
Enteritidis ABB07-SB3071 (#12)	p07-SB3071_1	IncF	57522	88	1	184
	p07-SB3071_2		5463	5	1	6128
	p07-SB3071_3		4634	7	1	3998
Enteritidis ABBSB1050-2 (#13)	pSB1050-2_1	IncI1	97,326	107	5	210
	pSB1050-2_2		1610	1	1	4090
	pSB1050-2_3		6647	7	1	1453
Enteritidis ABBSB1189-1 (#14)	pSB1189-1_1	IncF	93,837	134	1	N/A
	pSB1189-1_2	IncI1	97,892	110	1	N/A
Hadar ABB1048-1 (#15)	pABB1048-1_1		2286	3	1	10144
	pABB1048-1_2		16,874	26	1	386
	pABB1048-1_3		3904	3	1	7112
	pABB1048-1_4		119,773	103	1	285
Hadar ABBSB1020-2 (#16)	pSB1020-2_1	IncI1	81,744	82	9	275
	pSB1020-2_2		3894	3	1	2809
Hadar ABBSB1121-1 (#17)	pSB1121-1_1		5631	7	1	2358
	pSB1121-1_2		2110	3	1	6918
Heidelberg SALB-46 (#18)	pSALB-46_1	IncI1	92,304	106	5	315
	pSALB-46_2		37,685	48	2	484
	pSALB-46_3		3046	3	1	1906
Heidelberg SALB-159-4 (#19)	pSALB-159-4_1		37,685	48	2	543
	pSALB-159-4_2	IncI1	84,808	95	2	407
	pSALB-159-4_3		3046	3	1	1636
Heidelberg ABB07-SB3031 (#20)	p07-SB3031_1	IncI1	92,932	107	5	598
	p07-SB3031_2	IncQ	37,685	48	2	713
	p07-SB3031_3		2096	1	1	4971
Kentucky ABB1087 (#21)	pABB1087_1	IncF	55,928	51	3	328
	pABB1087_2		23,768	23	2	1718
	pABB1087_3		16,724	15	2	1245
	pABB1087_4		14,039	17	2	539
Kentucky SALC-205-3 (#22)	pSALC-205-3_1	IncI1	101,375	107	7	668
	pSALC-205-3_2		23,786	21	2	1576
	pSALC-205-3_3		12,970	13	2	1200
Kentucky ABB07-SB3057-2 (#23)	p07-SB3057-2_1	IncI1	75,167	77	7	665
	p07-SB3057-2_2		24,107	24	3	2051
	p07-SB3057-2_3		8290	9	3	1666
	p07-SB3057-2_4	IncF	109,020	112	12	377
Kentucky ABBSB1008-2 (#24)	pSB1008-2_1		22,770	19	3	1898
	pSB1008-2_2		12,552	10	3	1574
	pSB1008-2_3		6561	5	2	1271
	pSB1008-2_4	IncI1	90,890	97	5	894
	pSB1008-2_5		33,616	26	3	423
	pSB1008-2_6		24,872	18	4	586
Heidelberg SALB-47-2 (#25)	pSALB-47-2_1	IncI1	83,166	90	3	910
	pSALB-47-2_2		52,864	37	4	1154
	pSALB-47-2_3		11,868	4	1	2306

### Hypermutation-associated proteins

Clusters corresponding to *mutS*, *mutL* and *mutH* genes were predicted to be present in all isolates. MutS has a complex structure with an N-terminal mismatch recognition domain [49]; thus, truncated or deleted *mutS* genes in the above isolates could lead to an accumulation of mutations and acquired pathogenic phenotypes in these isolates. Poor read coverage around positions 1000–1200 in the *mutS* gene was noted in ABB07-SB3031 (serovar Heidelberg) and in two S. Kentucky (ABB1087-1 and SALC-205-3, which have a unique copper/silver efflux system). The protein clusters associated with methyl-directed repair DNA adenine methylase (DAM methylase) and ATP-dependent DNA helicase UvrD were found to be conserved in all 25 isolates. Isolates of serovar Heidelberg were found to carry an additional unique cluster which was also seen in Heidelberg reference strains.

## Discussion

This study compared genomes of 25 different isolates of *Salmonella enterica* belonging to the serovars Typhimurium, Enteritidis, Heidelberg, Hadar and Kentucky from commercial broiler farms in British Columbia, Canada. The genomic structure of these isolates is similar to that of reference *Salmonella* genomes selected from the database. As expected, all genomes were similar in their % G+C and gene density while the number of annotated protein-coding genes varied with the genome size. Our phylogenetic analysis revealed that apart from one isolate of serovar Enteritidis, all 24 other sequenced isolates clustered with isolates of the same serovar, confirming the genetic similarity of isolates from the same serovar used in this study. However, considerable variation in the presence and absence of specific genes and mobile elements was found.

Fimbriae and flagella play an essential function of colonization which is one of the first steps towards the onset of infection. Although flagella provide motility, adherence to cell surfaces is mainly mediated by fimbriae and other adherence-associated non-fimbrial proteins. There are approximately 13 known fimbrial gene operons in Typhimurium [50]. Although the clusters associated with all these operons were detected in sequenced *Salmonella*, only six operons (*fim*, *lpf*, *agf*/*csg*, *stf*, *sth* and *stb*) were found in all isolates. Additionally, two fimbrial operons, *pef* and *stc*, were also present in most *Salmonella*. Shippy et al. reported the protein StdA as a major contributor to the adhesion of *Salmonella* to the intestinal mucosa of poultry [51]. The truncated copy of this protein in serovar Hadar and S. Enteritidis ABBSB1050-2 suggests the presence of other possible adhesion mechanisms that could be involved in their ability to colonise the chicken gut. Furthermore, ShdA is an outer membrane protein of the auto-transporter family with fibronectin-binding ability that plays a possible role in the spread of *Salmonella enterica* subspecies to warm-blooded vertebrates [52]. The presence of this protein in only five isolates (all three Hadar, a Heidelberg and an Enteritidis) and its apparent lack in all four Kentucky and the remaining 16 isolates deserves additional investigation that would shed light on the role of this protein in the spread of specific *Salmonella* serovars in poultry and the consequent risks to humans. Kentucky also harbored a truncated flagellar hook protein FlgE. It should be noted that the adherence ability of our isolates to different tissues and matrices was not evaluated in the present study. Overall our data highlight the complexity of factors involved in the adherence of *Salmonella*.

Hemolysin E is a pore-forming toxin found in the family of *Enterobacteriacae* including *Salmonella* [37]. This hemolysin gene was completely absent from all 25 *Salmonella* genomes, however other hemolysin associated genes were detected in all of them. The operon *yej*ABEF encodes a putative ATP-binding cassette (ABC) transporter which contributes to the virulence of *Salmonella* by providing resistance to various host-produced antimicrobial peptides like protamine (from salmon sperm), melittin (from honey bee), and human β defensins (HBD)-1 [38] as well as polymyxin B (from *Paenibacillus polymyxa*). Gene clusters of the operon related to the resistance to antimicrobial peptides were detected in all our isolates suggesting that they could be involved in the persistence and/or virulence of our study serovar in chicken. However, the same YejABEF transporter is also associated with the uptake of and the sensitivity to microcin C, an antimicrobial peptide synthesized by the ribosome in some enterobacteria targeting an aminoacyl-tRNA synthetase [39]. Further investigation is warranted to understand the specific role(s) of this operon in order its exploitation as target in developing new therapeutic agents.

The T3SS plays a central role in *Salmonella*-host interaction and pathogenicity [53]. *Salmonella enterica* serovars possess two virulence-associated T3SSs, T3SS1 and T3SS2, in two distinct *Salmonella* pathogenicity islands, respectively SPI-1 and SPI-2. Although both SPIs have a similar set of genes encoding the T3SSs, a significant number of proteins encoded by either is unique to that particular SPI and may be associated with functions specific to its system. The effector molecules of SPI-1 trigger *Salmonella* invasion of the gut epithelial cells and enhance the colonization of the underlying tissues such as lamina propria. SPI-2 is essential to systemic infections and intracellular accumulation of *Salmonella* [54]. Many effectors secreted via T3SSs are also encoded outside these pathogenicity islands. For example, a significant role for the *sipA* gene (on SPI-1) and the *sop*ABDE2 operon (outside SPI-1) in *Salmonella* invasion of epithelial cells has been suggested [55]. The same effectors are also reported to be major virulence factors responsible for diarrhea in calves [56]. Several T3SS genes including the above-mentioned ones were detected in all 25 isolates.

Virulent and non-virulent strains are generally differentiated by a functional T3SS2 [57]. Figueira et al. showed that strains lacking the *sseG*, *sopD2* and *srfH* genes were attenuated in their virulence abilities although the strains retained intracellular vacuole integrity and a functional SPI-2 T3SS [58]. The lack of SPI2-associated genes SopD2, PipB2, SspH2 and SrfH in isolates of serovar Kentucky might explain in part their inability to induce diseases in humans. Despite variability observed in the T3SS component, conserved clusters of genes in this system were detected that could be used in designing effective strategies (diagnostic, vaccination or treatments) in the fight against *Salmonella*.

The horizontal transfer of genetic determinants from a donor bacterium into recipient bacteria is mediated by the type IV secretion system (T4SS) encoded on conjugative elements. The presence of several T4SS-associated genes in the corresponding isolates suggests the ability of these isolates to acquire and disseminate genetic determinants such as antibiotic resistance and virulence genes. Fourteen isolates were also carrying distinct T4SS-like conjugative IncF transfer systems including *S*. Kentucky ABB1087-1 and *S*. Kentucky ABB07-SB3057-2. It is interesting to note here that the corresponding two Kentucky isolates were also carrying unique iron acquisition features including siderophore aerobactin, salmochelin and operon *sitABCD* (discussed below) on a plasmid.

Iron availability is crucial for pathogenesis of *Salmonella* and an efficient iron uptake system is required for host adaptation [59]. Around 7% of *Salmonella* genes are known to be regulated by iron concentration [60]. Synthesis of aerobactin is considered to be an important contributor towards virulence of extracellularly growing *E*. *coli* and to the extracellular stages of growth of intracellular pathogens like *Shigella* [61]. Furthermore, the putative iron transport system *sitABCD* is also known to be involved in the virulence of *Salmonella* [62]. As they are also found in pathogenic bacteria, these above genes are important virulence factors whose presence in two of our Kentucky isolates deserves attention [14]. Accordingly, an *S*. *enterica* serovar Kentucky isolate associated with human infection has been reported in Europe [63]. All isolates contained Ton system-associated proteins TonB, ExbB and ExbD, which are required for the translocation of ferric siderophores into the periplasm [64]. FhuBCD, the ABC transporter complex associated with hydroxamate siderophore transport [65], was also present in all studied *Salmonella* isolates. Despite the wide spread detection of serovar Kentucky in North American poultry chicken and retail meats, salmonellosis cases associated with multidrug resistant Kentucky in Canada have been related to travel in Europe and Africa [66]. Our findings suggest that some *Salmonella* isolates belonging to serovar Kentucky not yet pathogenic in humans, unlike their European counterparts. These might evolve after acquisition of resistance and virulence genes, leading to the emergence of pathogenic multi-drug resistant Kentucky strains. Comparison studies between Kentucky isolated from Canadian broilers and those isolated from Europe and human infection cases in Canada are warranted.

The hypermutator phenotype is most often associated with defects in the methyl mismatch repair (MMR) system in Gram-negative bacteria. The primary role of this system is to execute a second level of proofreading to correct any mismatched or unpaired bases not corrected by the DNA polymerase-mediated proofreading activity [67,68]. MutS is a modulator protein which identifies the mismatch and initiates the cascade of repair mechanisms by the recruitment of MutL and endonuclease MutH. In the present work S. Heidelberg ABB07-SB3031, S. Kentucky isolates SALC-205-3 and ABB1087-1 were found to possess a possibly truncated *mutS* gene.

Although all the *Salmonella* were well equipped with multiple antibiotic resistance regulons and several efflux pumps, serovar Kentucky had the highest number of gene clusters associated with antibiotic and metal resistance group with some unique features such as arsenic resistance. In particular *S*. Kentucky SALC-205-3 also had gene clusters to confer copper and silver resistance. Among other serovars, Heidelberg carried a novel cluster to confer fosfomycin resistance which has not been seen in any *Salmonella*.

Although alignment with closed reference genomes showed a great deal of sequence order conservation, all of the newly sequenced genomes had some multiple contigs and reads that could not be assembled, due in part to our use of short Illumina reads. Similarly, while the plasmid identification approaches revealed a number of strong candidates with perfect or near-perfect tiling against reference plasmids, any plasmids in the newly sequenced genomes that had no corresponding plasmid in the reference database, and could not be distinguished from the main chromosome by read depth (e.g., single-copy plasmids) would not be identified using our approach. Similarly, our inferred plasmids spanned multiple contigs, thus raising the possibility that linking reads or short contigs may have been missed, and some short contigs included by mistake. Correcting and finishing plasmids would require a combination of sequencing techniques, such as long-read sequencing or generation of widely spaced mate-pair reads.

## Conclusion

Compared to the reference Salmonella found in the database, our studied broiler Salmonella isolates possessed some unique characteristics. The variations in gene content such as those for adhesins, flagella, iron acquisition, T3SS, T4SS and drug resistance, observed among the study isolates highlights the diversity of *Salmonella* serovars found in broiler chicken. The present study also indicates the presence of various genetic factors contributing to the survival and virulence of *Salmonella* in chicken gut that can also contribute to their characteristics as a foodborne pathogen. In addition to those previously reported [6], whole-genome sequencing revealed the presence of antimicrobial resistance determinants of transferable mobile genetic elements in most of our study isolates. The potential transfer of antimicrobial resistance determinants from farms to humans through enteric bacterial floras is a major food safety concern. Furthermore, our findings showed the presence of several chromosome-encoded efflux pumps in the studied *Salmonella* that would also contribute to their multidrug resistance and survival in hostile environments. Data from the present study could be useful for the control of *Salmonella* in broiler chickens such as identification of diagnostic, vaccine or antimicrobial targets. Further studies are needed to better understand the biology, evolution and survival of *Salmonella* serovars from poultry and poultry products.

## Supporting Information

S1 Fig (a) Plot of n50 values for each genome, for values of *k* between 43 and 51 inclusive (b) Plot of n50 versus average base quality for 24 *Salmonella* genomes sequenced using the Illumina HiSeq platform.
(PDF)

S2 Fig Distribution of each of the 9423 inferred protein clusters in the 25 newly sequenced *Salmonella* genomes. Clusters can be represented in one (cluster is unique to a single genome) to 25 (cluster is represented in all genomes) of the sequenced isolates.
(PNG)

S3 Fig Phylogenetic tree of all sequenced *Escherichia* and *Salmonella* isolates using the concatenated ribosomal genes with RAxML version 7.2.5-mpi. The *Escherichia coli* clade is collapsed into a single branch. Numbers at internal nodes correspond to bootstrap support values. *** indicates the 25 newly sequenced *Salmonella* genomes of this study.
(PNG)

S4 Fig Heatmap of all matches to MVirDB as shown in Fig 7, with cluster and gene names added. Another 111 ubiquitous proteins are not shown. Labels and colors are consistent with Fig 2.
(PNG)

S1 File Archive of multiple FASTA files containing unique cluster identifiers and sequences from each cluster reported in the text. (ZIP)

S2 File Complete phylogenetic tree in Newick format, containing all genomes used in the study and associated bootstrap values. Newly sequenced isolates are indicated with asterisks.
(ZIP)

S1 Table Assembly statistics for genomes sequenced in this study. (XLSX)

S2 Table Summary of inferred protein clusters, with unique ID, patterns of presence and absence across genomes from *Escherichia* and *Salmonella* isolates, aggregated names of homologous matches from these two genera, and statistics on the minimum, mean and maximum cluster length. (XLSX)

S3 Table Detailed list of plasmids showing names, predicted plasmid size, read depth of associated contigs, and predicted gene names based on homology searches. (XLSX)
