# Correction: Genomic Comparison of Non-Typhoidal *Salmonella enterica* Serovars Typhimurium, Enteritidis, Heidelberg, Hadar and Kentucky Isolates from Broiler Chickens

**DOI:** 10.1371/journal.pone.0137697

**Published:** 2015-09-09

**Authors:** Akhilesh S. Dhanani, Glenn Block, Ken Dewar, Vincenzo Forgetta, Edward Topp, Robert G. Beiko, Moussa S. Diarra

The bottom of Table 1 is missing four isolates. The authors have provided a corrected version of Table 1 below.

**Table 1:** List of the *Salmonella enterica* serovars from 15 different farms and their relevant characteristics as previously described.

Genome	Inventory #	*S*. *enterica* Serovar	Isolate	Sampling date	Farm	Age (days)	Resistance spectrum	Resistance genes	Virulence genes
**1**	3198	Typhimurium	ABBSB1113-1	26-Jun-2006	N	38	*/*	*/*	*invA*
**2**	1813	Typhimurium	SALF-297-3	Dec 6 2004	F	28	*/*	*strA*	*invA-spvC*
**3**	1893	Typhimurium	SALH-394-2	Dec 20 2004	H	12	AC,T,AM,F,C,Su,Te/Ax	*blaCMY2-strA-aadA1*	*invA-spvC*
**5**	1812	Typhimurium	SALF-297-2	Dec 6 2004	F	28	*/*	*strA*	*invA-spvC*
**8**	1803	Typhimurium	SALF-276-1	Nov 13 2004	F	12	AC,T,AM,F/Ax	*strA*	*invA-spvC*
**9**	3166	Typhimurium	ABB1162-2	27-Jun-2005	ND	ND	AC,T,AM,F,G,K,Su,Te	*tetB-strA-sul1*	*invA*
**10**	3148	Typhimurium	ABBSB1116-2	27-Jun-2006	O	36	*/*	*/*	*invA*
**4**	3176	4,[5],12:i:	ABBSB1205-1	2-Aug-2006	R	40	AC,T,AM,F/Ax	*blaCMY2*	*invA-spvC*
**6**	2008	4,[5],12:i:	SALI-436-2	Mar 3 2005	I	10	*/*	*strA-aadA1*	*invA-spvC*
**7**	3128	4,[5],12:i:	ABBSB1218-1	10-Aug-2006	S	42	AC,T,AM,F/Ax	*blaCMY2-blaTEM*	*invA-spvC*
**11**	3180	Enteritidis	ABBSB1004-1	9-May-2006	J	32	*/*	*/*	*invA*
**12**	3346	Enteritidis	ABB07-SB3071	28-Jun-2005	ND	ND	*/*	*/*	*invA-spvC*
**13**	3193	Enteritidis	ABBSB1050-2	29-May-2006	M	32/34	AC,T,AM,F	*blaCMY2-blaTEM*	*invA*
**14**	3172	Enteritidis	ABBSB1189-1	26-Jul-2006	M	33	AC,T,AM,F/Ax	*blaCMY2-blaTEM*	*invA-spvC*
**15**	3137	Hadar	ABB1048-1	27-Jun-2005	ND	ND	S,Te,	*tetA-tetB-strA*	*invA*
**16**	3187	Hadar	ABBSB1020-2	16-May-2006	L	42	AC,T,AX,AM,F,S,Su,Te	*blaCMY2-blaTEM-tetA-tetB-aadA1-aadA2-sul1-strA*	*invA*
**17**	3147	Hadar	ABBSB1121-1	28-Jun-2006	P	37	AM,T,Ax,Su,TSx,	*sul1*	*invA*
**18**	1770	Heidelberg	SALB-46	Oct 4, 2004	B	10	AC,T,AP,F,Ax	*strA*	*invA*
**19**	1773	Heidelberg	SALB-159-4	Oct 25, 2004	B	28	AC,T,AP,F,Ax	*blaCMY2-strA*	*invA*
**20**	3342	Heidelberg	ABB07-SB3031	28-Jun-2005	ND	ND	AC,T,AM,F,Ax	*blaCMY2-blaTEM*	*invA*
**25**	1768	Heidelberg	SALB-47-2	Oct 4, 2004	B	10	AC,T,AM,F,Ax	*strA*	*invA*
**21**	3144	Kentucky	ABB1087-1	27-Jun-2005	ND	ND	AC,T,AM,F,Ax,te	*tetB*	*invA*
**22**	1787	Kentucky	SALC-205-3	Nov 1 2004	C	28	*/*	*strA*	*invA*
**23**	3313	Kentucky	ABB07-SB3057-2	28-Jun-2005	ND	ND	AC,T,AM,F,Ax,S,Su,Te	*blaSHV-blaTEM-tetB-strA-sul1*	*invA*
**24**	3184	Kentucky	ABBSB1008-2	10-May-2006	K	43	AC,T,AM,F,Ax	*blaSHV*	*invA*

In the Materials and Methods section beneath the header “Identification of functional gene classes,” the subheader “Iron resistance proteins” should instead be “Iron regulated proteins.”
