# Correction: Genomic Comparison of Non-Typhoidal Salmonella enterica Serovars Typhimurium, Enteritidis, Heidelberg, Hadar and Kentucky Isolates from Broiler Chickens

**DOI:** 10.1371/journal.pone.0148706

**Published:** 2016-02-08

**Authors:** Akhilesh S. Dhanani, Glenn Block, Ken Dewar, Vincenzo Forgetta, Edward Topp, Robert G. Beiko, Moussa S. Diarra

There are errors in the published article. The isolate # 3172 ABBSB1189-1 (genome 14) was named as Enteritidis by mistake. The correct isolate should be Typhimurium.

Serotyping was incorrect for the isolate # 3193 ABBSB1050-2 (genome 13). It was incorrectly labeled as Enteritidis. The correct label should be Thompson.

These errors affect Figs 1C and 2–7, S3 and S4 Figs, and S1 and S2 Tables. Please view correct versions of the files here.

**Fig 1:**
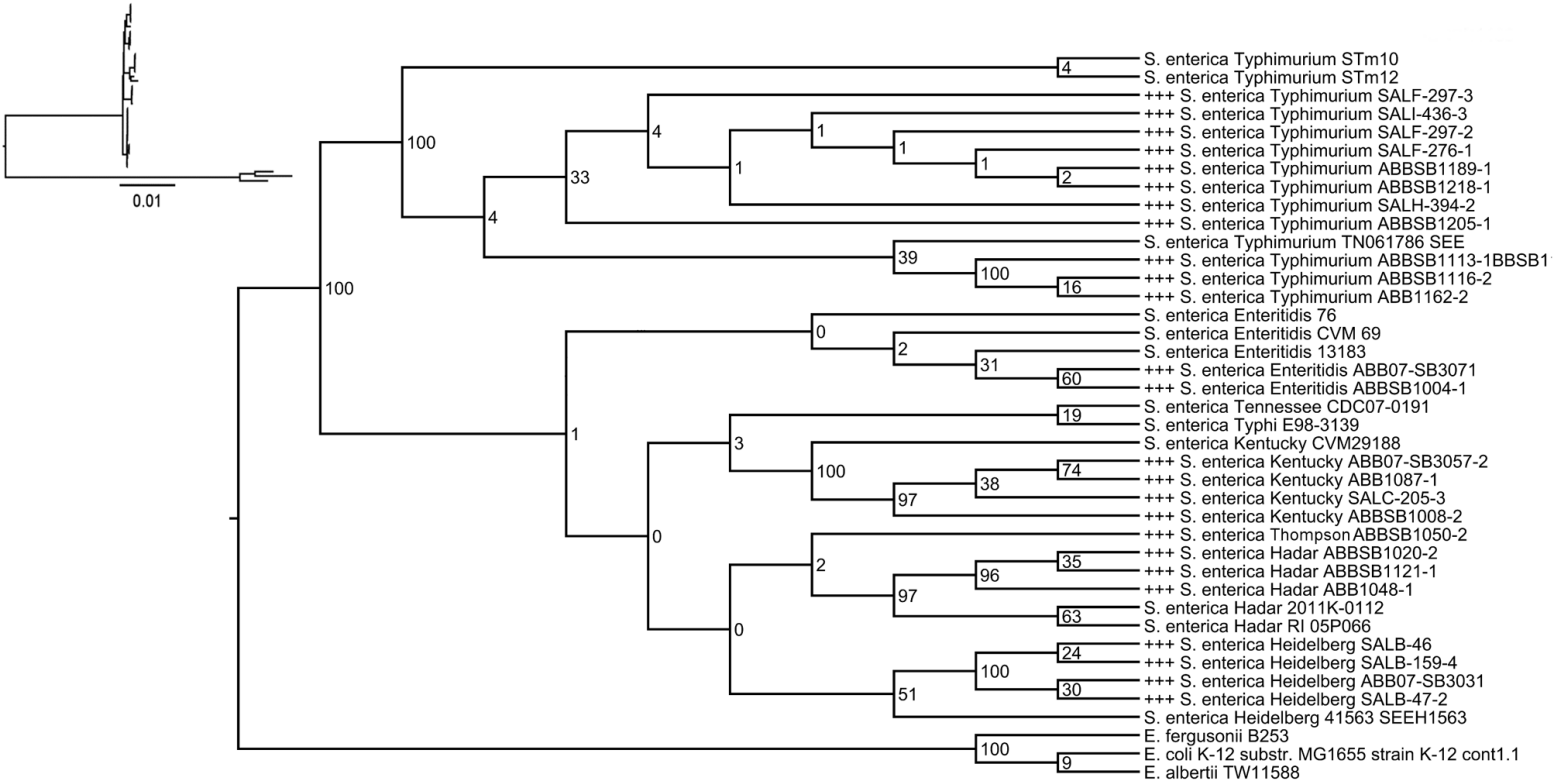
Projected phylogeny of isolate genomes extracted from the full tree of *Escherichia* and *Salmonella* isolates (see S2 Fig). Numbers at internal nodes correspond to bootstrap values supporting the indicated clades in the full tree. “+++” indicates newly sequenced isolates. Inset: pruned tree shown with branch lengths corresponding to substitutions per site.

**Fig 2:**
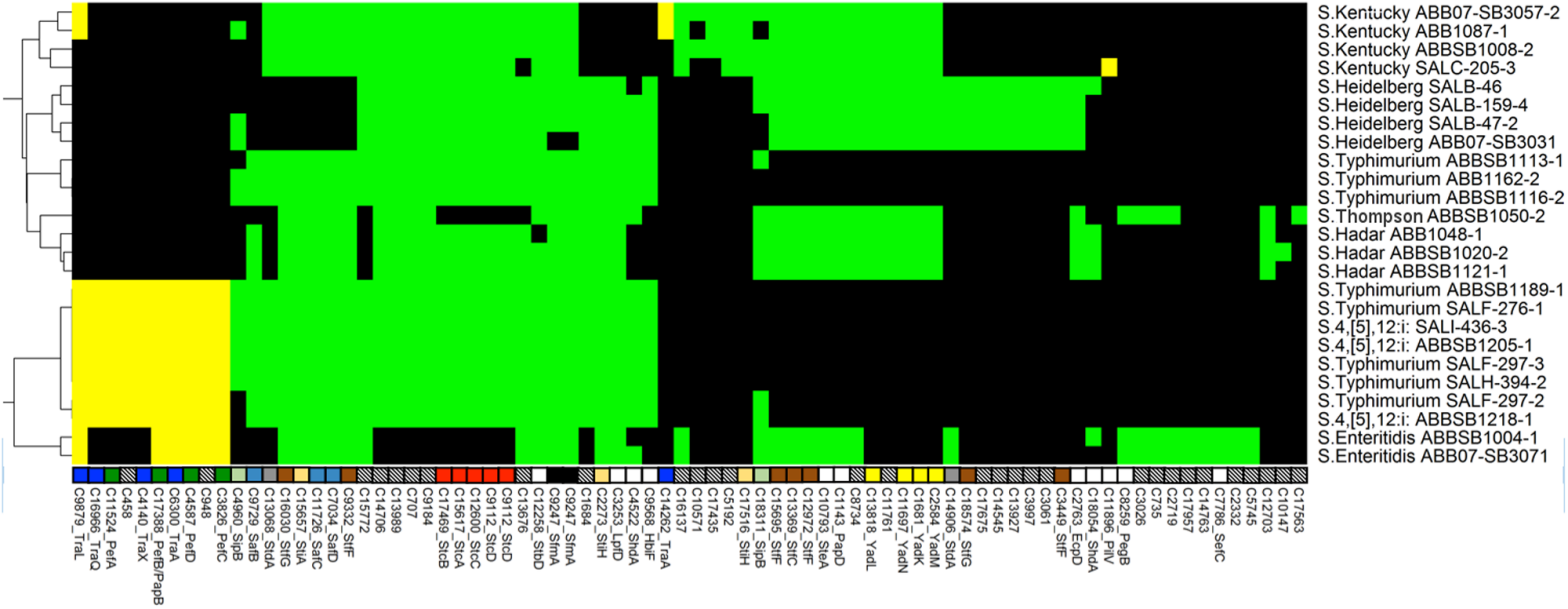
Heatmap showing the distribution of 78 adhesion-associated proteins across the 25 newly sequenced *Salmonella*genomes. Another 54 ubiquitous proteins are not shown. Column labels indicate unique cluster IDs (see S1 File), and gene names where appropriate. Black cells = absence of a gene from a given strain, green cells = probable chromosomal, yellow = probable plasmid. The tree on the left-hand side of the figure shows a hierarchical complete-linkage clustering of the profiles based on Euclidean distance. Multiple genes affiliated with the same operon are identified by coloured square boxes above the cluster legends. Empty boxes represent a single, independent cluster and boxes with patterns represent clusters without annotated gene names.

**Fig 3:**
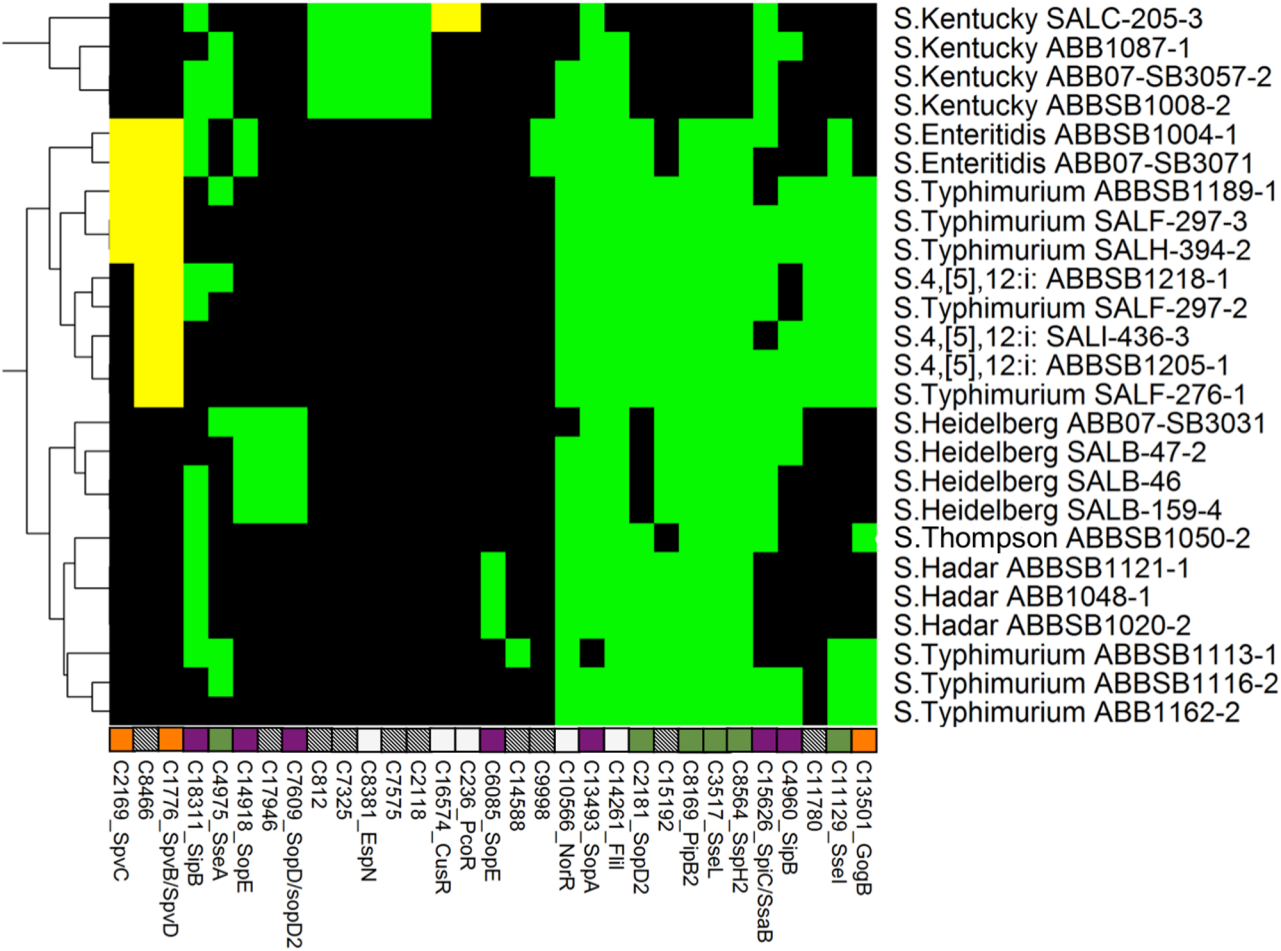
Heatmap showing the distribution of 31 type 3 secretion system proteins. Another 111 ubiquitous proteins are not shown. Labels, colors and clustering are consistent with Fig 2.

**Fig 4:**
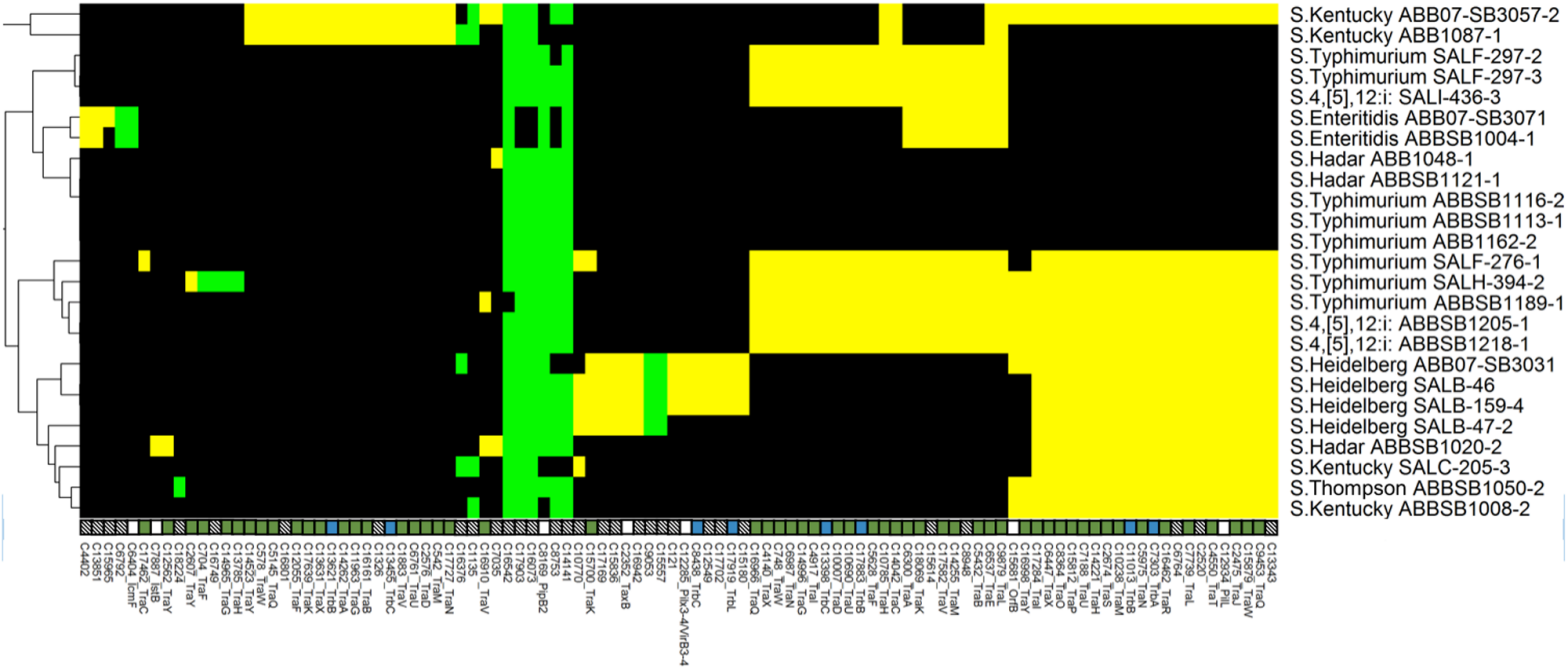
Heatmap showing the distribution of 102 T4SS proteins. Another 17 ubiquitous proteins are not shown. Labels, colors and clustering are consistent with Fig 2.

**Fig 5:**
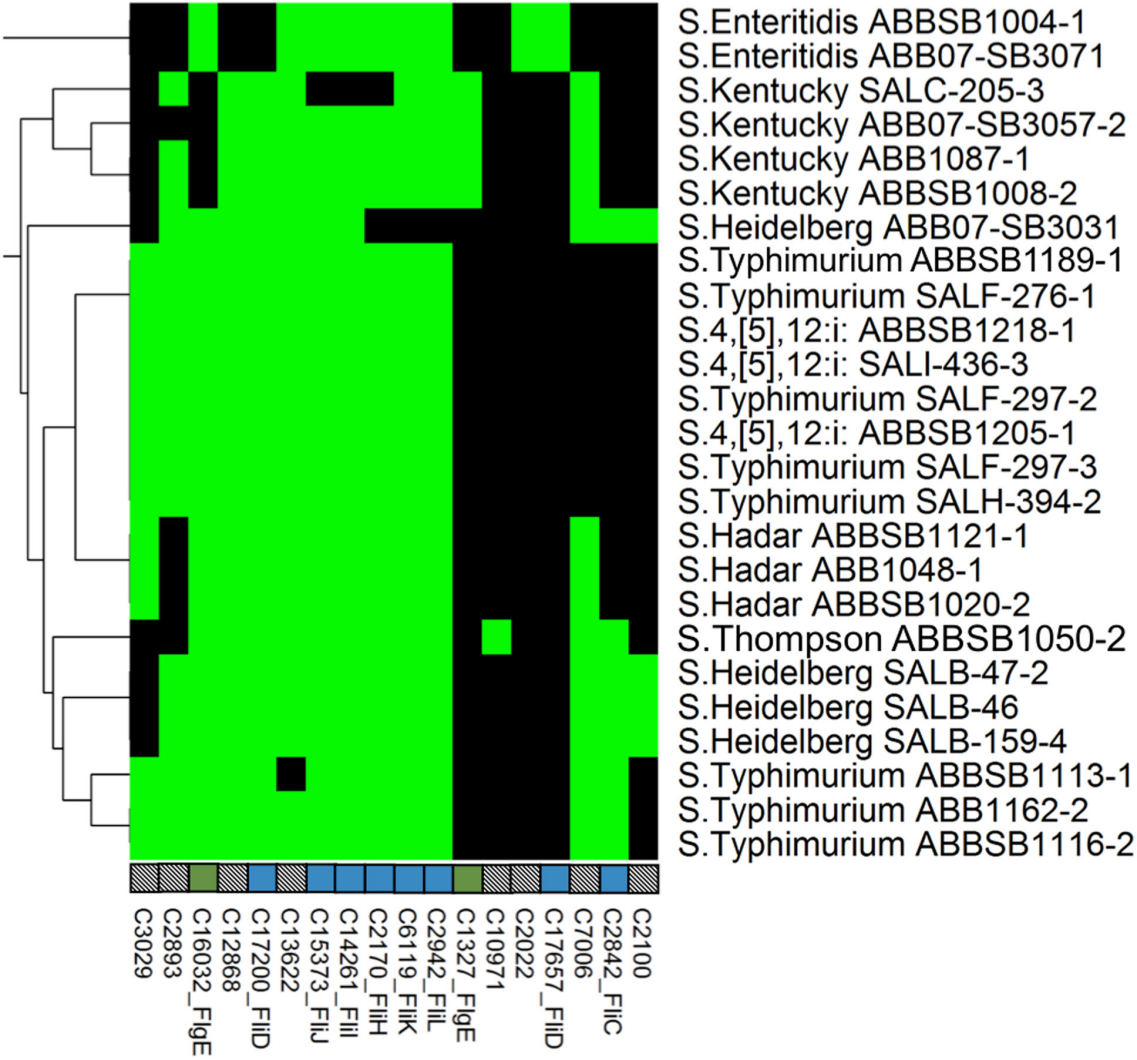
Heatmap showing the distribution of 18 flagellar proteins. Another 40 ubiquitous proteins are not shown. Labels, colors and clustering are consistent with Fig 2 (no flagellar proteins were predicted to be plasmid associated).

**Fig 6:**
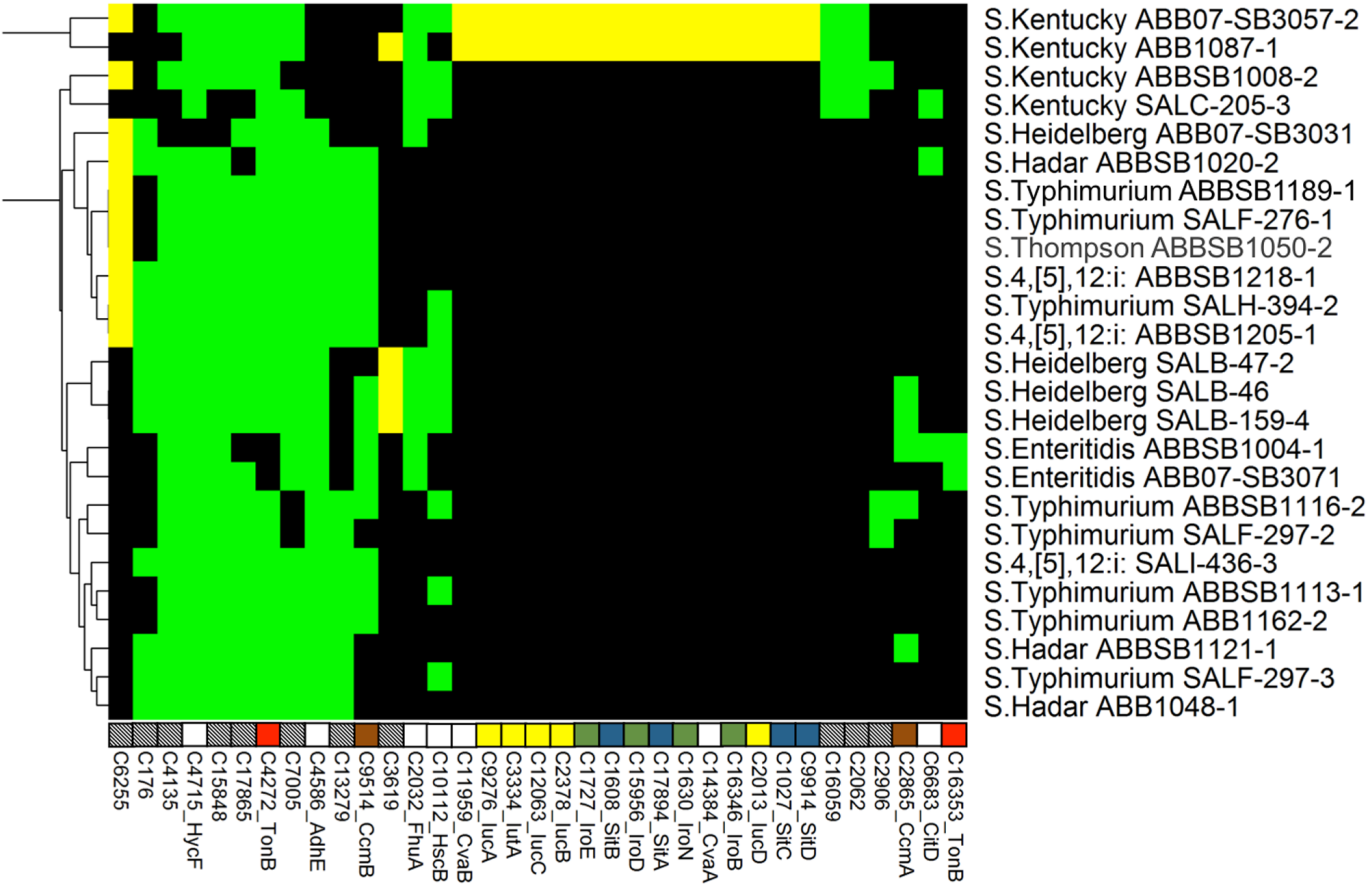
Heatmap showing the distribution of 35 iron resistance proteins. Another 87 ubiquitous proteins are not shown. Labels, colors and clustering are consistent with Fig 2.

**Fig 7:**
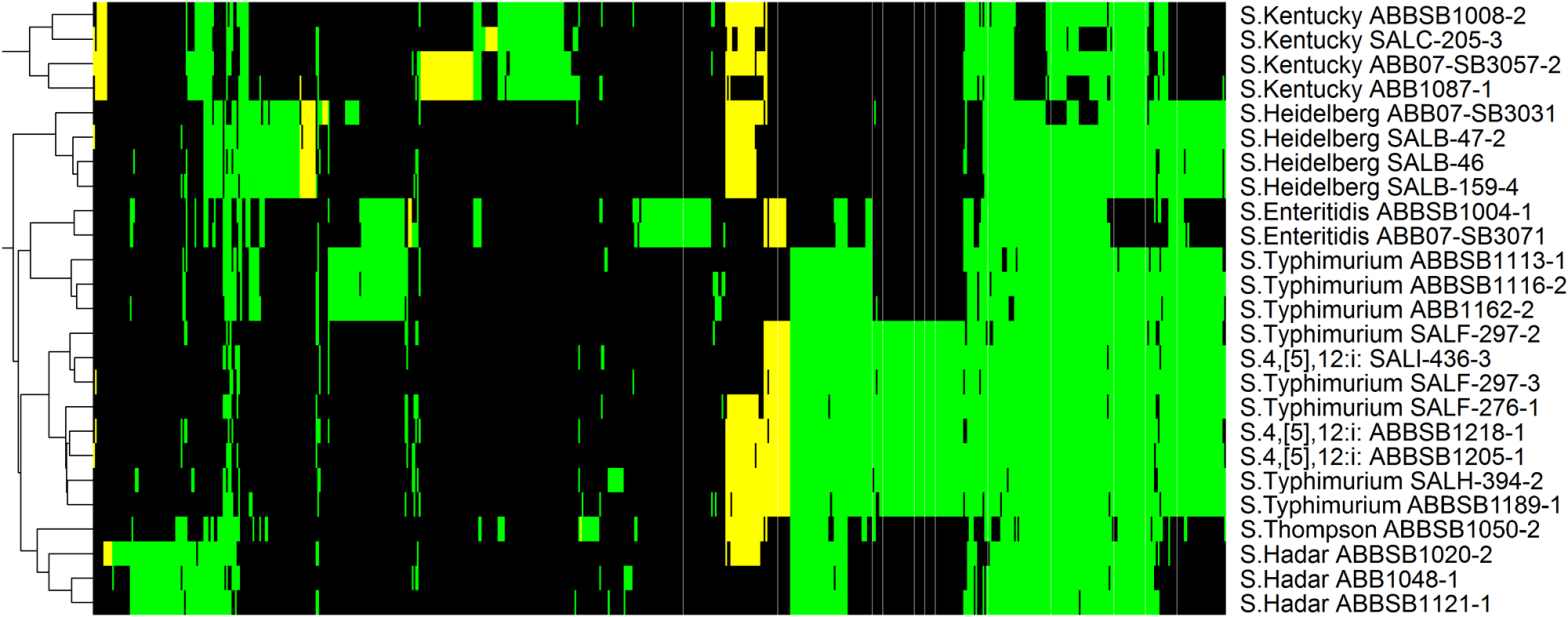
Heatmap of 647 proteins matching to the MVirDB database. Another 784 ubiquitous proteins are not shown. Colors are consistent with Fig 2; column labels are shown in S4 Fig.

## Supporting Information

S3 Fig Phylogenetic tree of all sequenced *Escherichia* and *Salmonella* isolates using the concatenated ribosomal genes with RAxML version 7.2.5-mpi. The *Escherichia coli* clade is collapsed into a single branch. Numbers at internal nodes correspond to bootstrap support values. *** indicates the 25 newly sequenced *Salmonella* genomes of this study.
(TIFF)

S4 Fig Heatmap of all matches to MVirDB as shown in Fig 7, with cluster and gene names added. Another 111 ubiquitous proteins are not shown. Labels and colors are consistent with Fig 2.
(TIFF)

S1 Table Assembly statistics for genomes sequenced in this study. (XLSX)

S2 Table Summary of inferred protein clusters, with unique ID, patterns of presence and absence across genomes from *Escherichia* and *Salmonella* isolates, aggregated names of homologous matches from these two genera, and statistics on the minimum, mean and maximum cluster length. (XLSX)
