# Supplementary figures and images for: Genomic Comparison of Non-Typhoidal Salmonella enterica Serovars Typhimurium, Enteritidis, Heidelberg, Hadar and Kentucky Isolates from Broiler Chickens
Source: PLoS One. 2015 Jun 17;10(6):e0128773. doi: 10.1371/journal.pone.0128773 (PMC4470630; doi:10.1371/journal.pone.0128773)

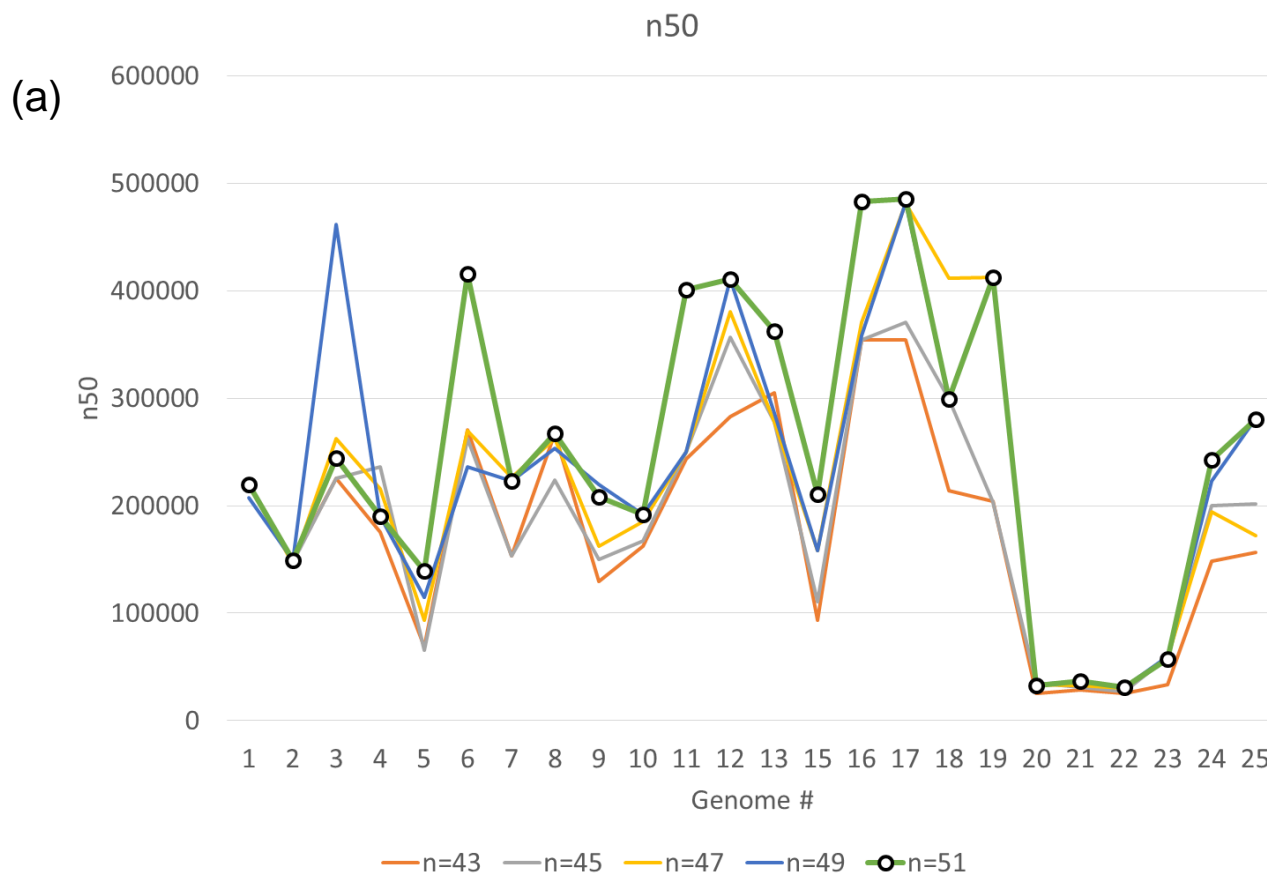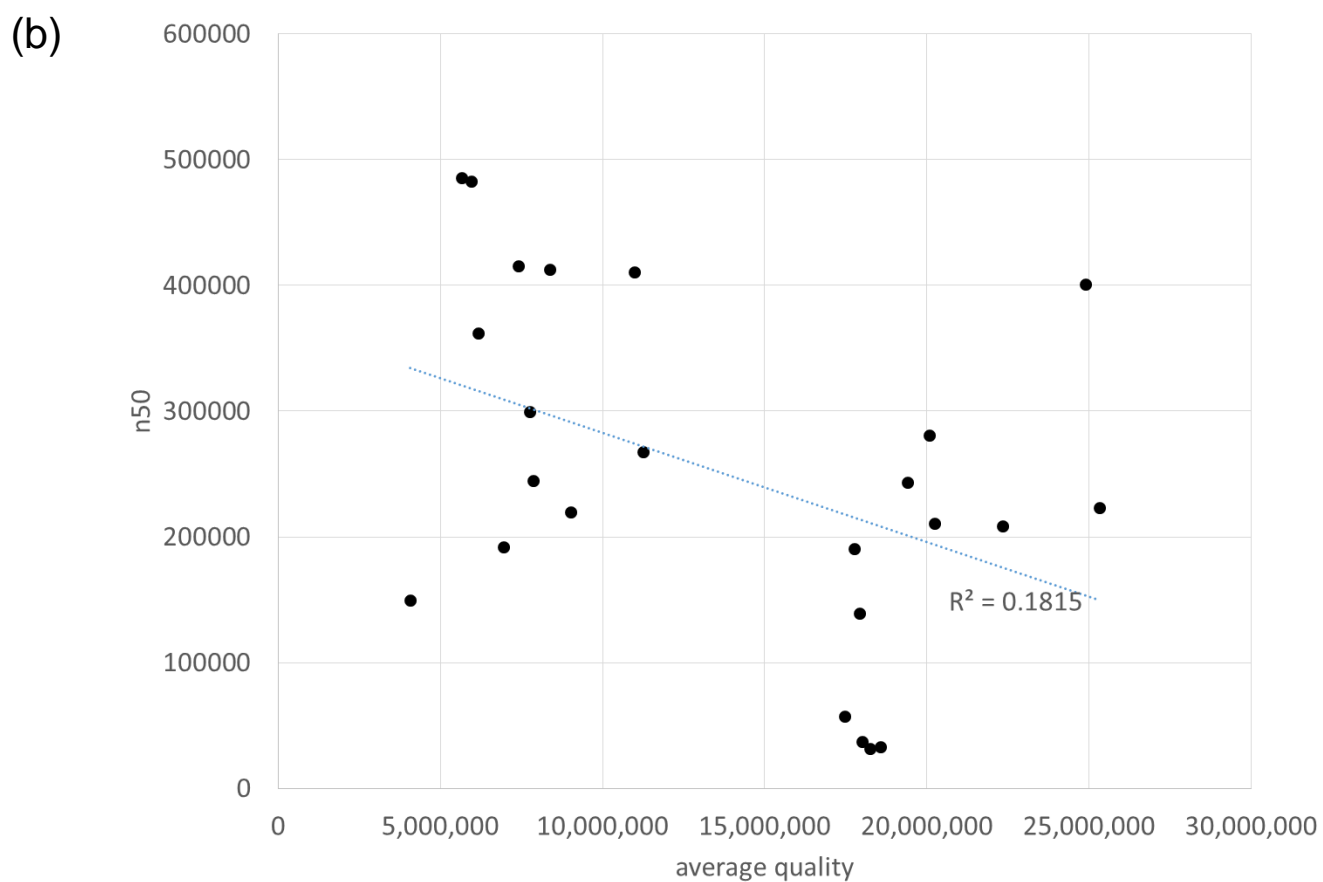

Supplement: S1 Fig — (a) Plot of n50 values for each genome, for values of k between 43 and 51 inclusive (b) Plot of n50 versus average base quality for 24 Salmonella genomes sequenced using the Illumina HiSeq platform. (PDF) [file pone.0128773.s001.pdf]

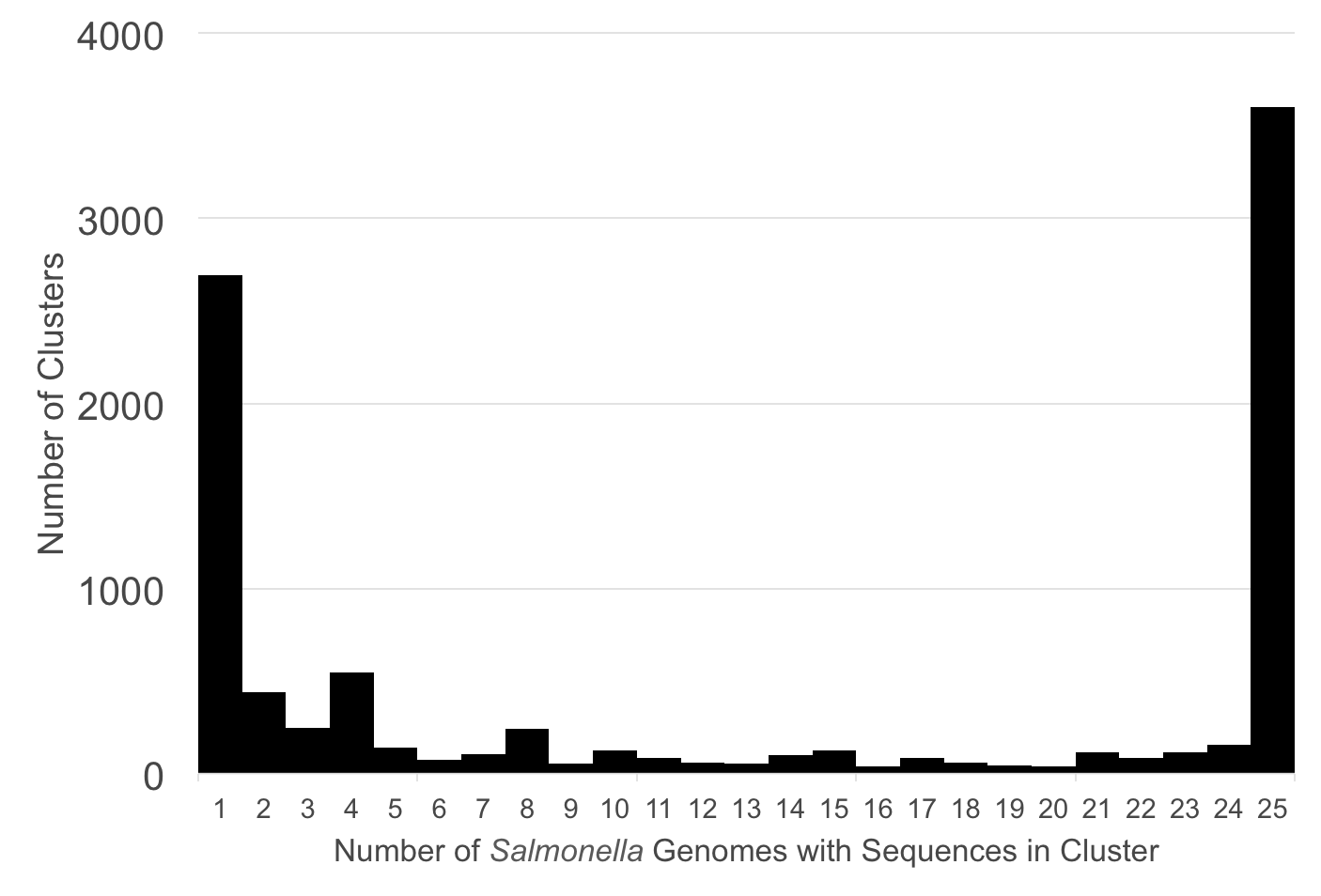

Supplement: S2 Fig — Clusters can be represented in one (cluster is unique to a single genome) to 25 (cluster is represented in all genomes) of the sequenced isolates. (PNG) [file pone.0128773.s002.png]

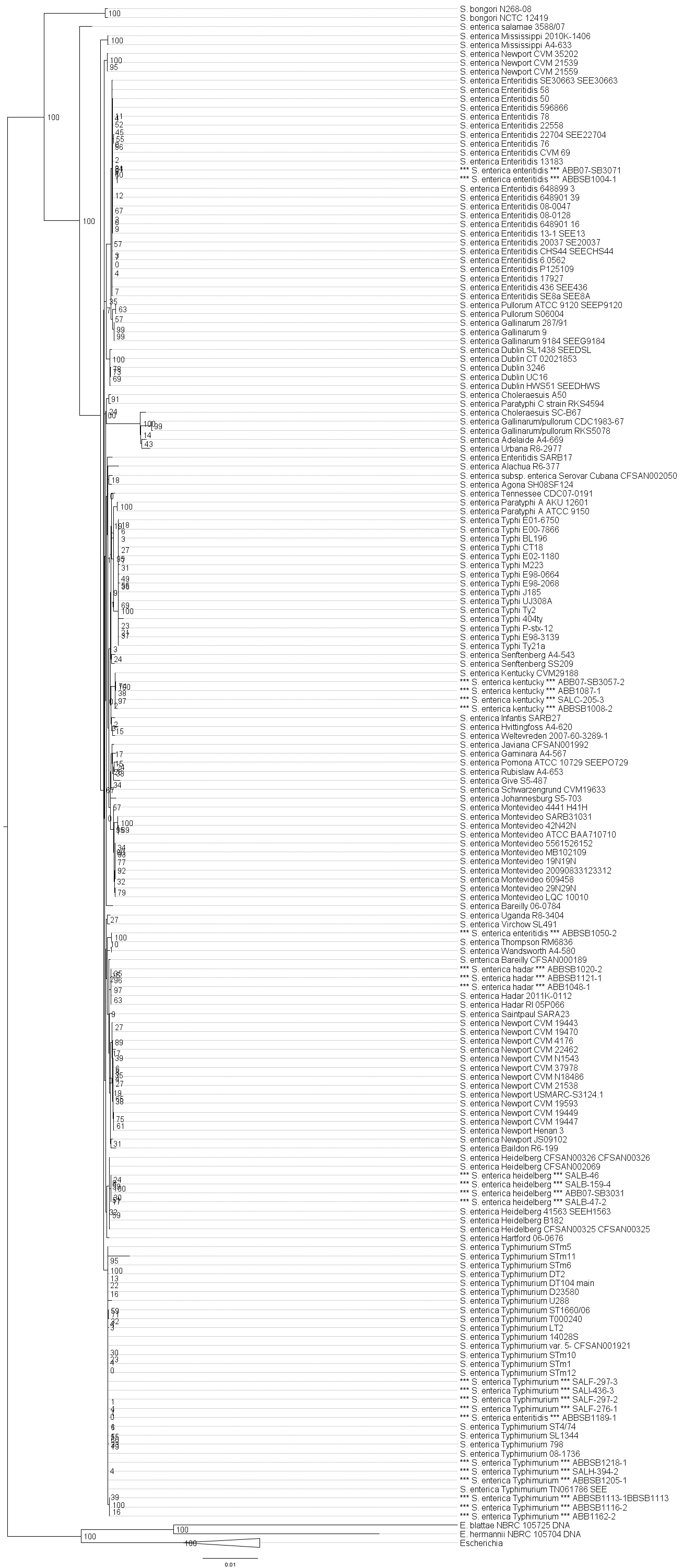

Supplement: S3 Fig — The Escherichia coli clade is collapsed into a single branch. Numbers at internal nodes correspond to bootstrap support values. *** indicates the 25 newly sequenced Salmonella genomes of this study. (PNG) [file pone.0128773.s003.png]
