# Supplementary figures and images for: Correction: Genomic Comparison of Non-Typhoidal Salmonella enterica Serovars Typhimurium, Enteritidis, Heidelberg, Hadar and Kentucky Isolates from Broiler Chickens
Source: PLoS One. 2016 Feb 8;11(2):e0148706. doi: 10.1371/journal.pone.0148706 (PMC4746026; doi:10.1371/journal.pone.0148706)

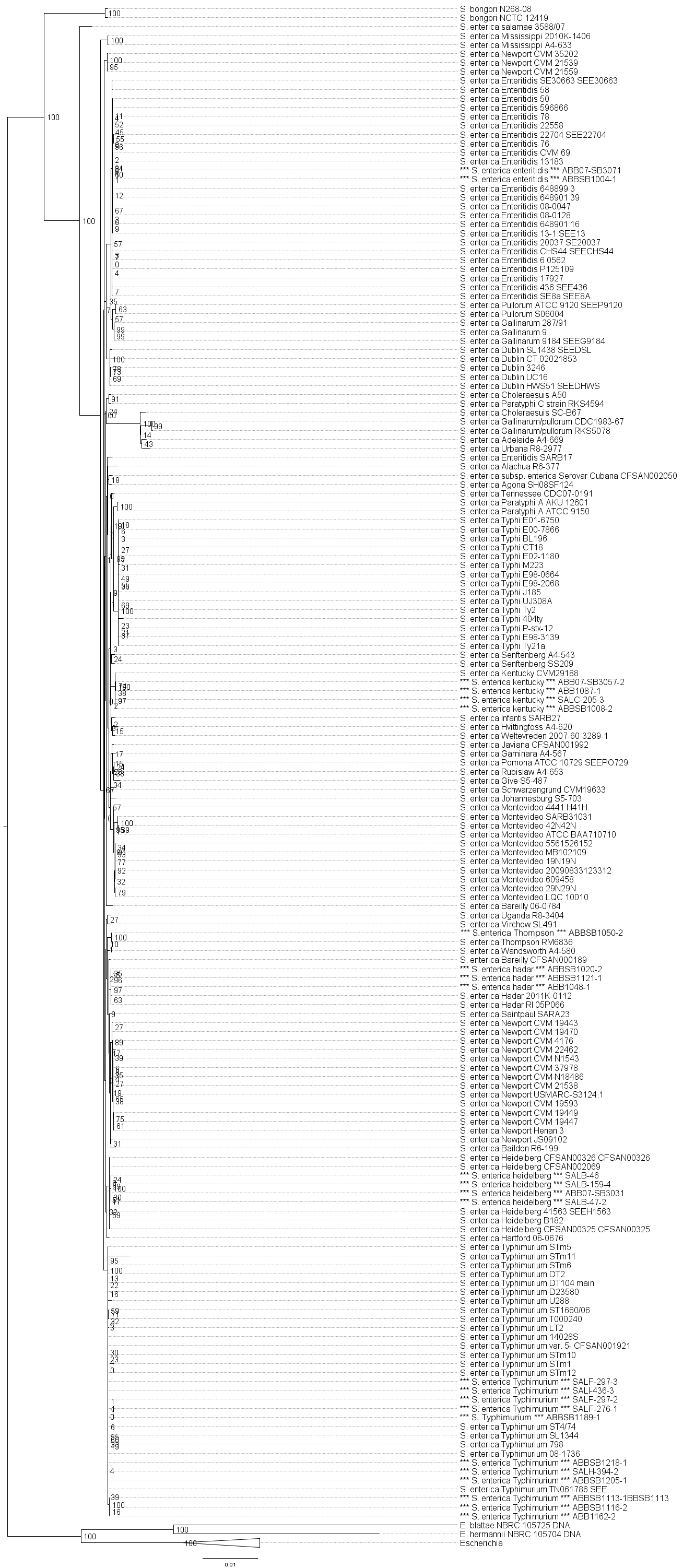

Supplement: S3 Fig — The Escherichia coli clade is collapsed into a single branch. Numbers at internal nodes correspond to bootstrap support values. *** indicates the 25 newly sequenced Salmonella genomes of this study. (TIFF) [file pone.0148706.s001.tiff]
